# Supplementary material for: Medical imaging utilization in migrants compared with nonmigrants in a universal healthcare system: A population-based matched cohort study
Source: PLoS Med. 2024 Oct 22;21(10):e1004474. doi: 10.1371/journal.pmed.1004474 (PMC11495850; doi:10.1371/journal.pmed.1004474)
Supplement: S1 Checklist — (PDF) [file pmed.1004474.s001.pdf]

S1 Checklist. STROBE Statement–Checklist of items that should be included in reports of cohort studies

	Item No.	Recommendation	Page No.
**Title and abstract**	1	(a) Indicate the study's design with a commonly used term in the title or the abstract	Title/Abstract
		(b) Provide in the abstract an informative and balanced summary of what was done and what was found	Abstract
**Introduction**			
Background / rational	2	Explain the scientific background and rationale for the investigation being reported	Introduction, para 1-4
Objectives	3	State specific objectives, including any prespecified hypotheses	Introduction, para 5
**Methods**			
Study design	4	Present key elements of study design early in the paper	*Population and study design*, para 1-2
Setting	5	Describe the setting, locations, and relevant dates, including periods of recruitment, exposure, follow-up, and data collection	*Population and study design*, para 1
Participants	6	(a) Give the eligibility criteria, and the sources and methods of selection of participants. Describe methods of follow-up	*Population and study design*, para 1-2
		(b) For matched studies, give matching criteria and number of exposed and unexposed	
Variables	7	Clearly define all outcomes, exposures, predictors, potential confounders, and effect modifiers. Give diagnostic criteria, if applicable	*Imaging utilization and covariates*, para 1-2
Data sources / measurement	8	For each variable of interest, give sources of data and details of methods of assessment (measurement). Describe comparability of assessment methods if there is more than one group	*Data sources*, para 1-3
Bias	9	Describe any efforts to address potential sources of bias	*Statistical analysis*, para 2-4; *Sensitivity analyses*
Study size	10	Explain how the study size was arrived at	*Population and study design*, para 1-2
Quantitative variables	11	Explain how quantitative variables were handled in the analyses. If applicable, describe which groupings were chosen and why	*Statistical analysis*, para 1,4
Statistical methods	12	(a) Describe all statistical methods, including those used to control for confounding	*Statistical analysis*, para 1-4
		(b) Describe any methods used to examine subgroups and interactions	
		(c) Explain how missing data were addressed	
		(d) If applicable, explain how loss to follow-up was addressed	
		(e) Describe any sensitivity analyses	*Sensitivity analyses*
**Results**			

Participants	13	(a) Report numbers of individuals at each stage of study—eg numbers potentially eligible, examined for eligibility, confirmed eligible, included in the study, completing follow-up, and analysed	Results, para 1
		(b) Give reasons for non-participation at each stage	
		(c) Consider use of a flow diagram	
Descriptive data	14	(a) Give characteristics of study participants (eg demographic, clinical, social) and information on exposures and potential confounders	Table 1
		(b) Indicate the number of participants with missing data for each variable of interest	
		(c) Summarize baseline characteristics	
Outcome data	15	Report numbers of outcome events or summary measures over time	Fig1, Fig3, S1, S2, S3 Table
Main results	16	(a) Give unadjusted estimates and, if applicable, confounder-adjusted estimates and their precision (eg, 95% confidence interval). Make clear which confounders were adjusted for and why they were included	Table 2; *Multivariable recurrent event model of imaging*, para 1-3
		(b) Report category boundaries when continuous variables were categorized	
		(c) If relevant, consider translating estimates of relative risk into absolute risk for a meaningful time period	
Other analyses	17	Report other analyses done—eg analyses of subgroups and interactions, and sensitivity analyses	*Imaging utilization for time since immigration*, para 1-3
**Discussion**			
Key results	18	Summarize key results with reference to study objectives	Discussion, para 1-2, 5-6
Limitations	19	Discuss limitations of the study, taking into account sources of potential bias or imprecision. Discuss both direction and magnitude of any potential bias	Discussion, para 10
Interpretation	20	Give a cautious overall interpretation of results considering objectives, limitations, multiplicity of analyses, results from similar studies, and other relevant evidence	Discussion, para 3-7
Generalizability	21	Discuss the generalizability (external validity) of the study results	Discussion, para 3-7
**Other information**			
Funding	22	Give the source of funding and the role of the funders for the present study and, if applicable, for the original study on which the present article is based	*Funding*
