# Supplementary material for: Medical imaging utilization in migrants compared with nonmigrants in a universal healthcare system: A population-based matched cohort study
Source: PLoS Med. 2024 Oct 22;21(10):e1004474. doi: 10.1371/journal.pmed.1004474 (PMC11495850; doi:10.1371/journal.pmed.1004474)
Supplement: S1 Table — (PDF) [file pmed.1004474.s002.pdf]

S1 Table. Mean cumulative number of imaging exams every 5-years of follow-up stratified by age at migration.

	Migrant				Non-migrant			
**Modality**	**5th year**	**10th year**	**15th year**	**20th year**	**5th year**	**10th year**	**15th year**	**20th year**
0-19 years at index								
Computerized tomography	0.06 (0.05, 0.06)	0.14 (0.14, 0.14)	0.26 (0.26, 0.26)	0.42 (0.41, 0.42)	0.08 (0.08, 0.08)	0.19 (0.19, 0.20)	0.35 (0.35, 0.36)	0.56 (0.55, 0.56)
Magnetic resonance imaging	0.03 (0.03, 0.03)	0.09 (0.09, 0.10)	0.20 (0.20, 0.20)	0.35 (0.35, 0.35)	0.05 (0.05, 0.05)	0.14 (0.14, 0.14)	0.29 (0.28, 0.29)	0.48 (0.48, 0.49)
Radiography	0.96 (0.95, 0.96)	1.99 (1.98, 2.00)	3.08 (3.07, 3.10)	4.23 (4.20, 4.25)	1.25 (1.25, 1.26)	2.57 (2.56, 2.58)	3.94 (3.92, 3.96)	5.33 (5.30, 5.36)
Ultrasound	0.39 (0.39, 0.40)	1.09 (1.08, 1.09)	2.21 (2.20, 2.23)	3.88 (3.85, 3.91)	0.39 (0.39, 0.39)	1.06 (1.05, 1.06)	2.19 (2.18, 2.21)	3.94 (3.91, 3.98)
20-39 years at index								
Computerized tomography	0.16 (0.16, 0.16)	0.38 (0.38, 0.38)	0.66 (0.66, 0.67)	1.02 (1.01, 1.02)	0.22 (0.21, 0.22)	0.50 (0.49, 0.50)	0.86 (0.86, 0.87)	1.34 (1.33, 1.35)
Magnetic resonance imaging	0.09 (0.09, 0.09)	0.24 (0.24, 0.24)	0.47 (0.47, 0.47)	0.75 (0.75, 0.76)	0.14 (0.14, 0.14)	0.36 (0.36, 0.36)	0.67 (0.67, 0.68)	1.07 (1.07, 1.08)
Radiography	1.47 (1.47, 1.48)	3.16 (3.15, 3.17)	5.20 (5.18, 5.22)	7.69 (7.66, 7.72)	1.62 (1.61, 1.63)	3.54 (3.53, 3.55)	5.99 (5.97, 6.01)	9.06 (9.02, 9.09)
Ultrasound	2.50 (2.49, 2.51)	4.87 (4.85, 4.89)	7.00 (6.98, 7.03)	9.07 (9.04, 9.10)	2.08 (2.07, 2.09)	4.25 (4.23, 4.27)	6.25 (6.22, 6.27)	8.16 (8.13, 8.20)
40-59 years at index								
Computerized tomography	0.33 (0.32, 0.33)	0.77 (0.76, 0.77)	1.34 (1.33, 1.35)	2.07 (2.05, 2.09)	0.47 (0.46, 0.47)	1.07 (1.06, 1.08)	1.87 (1.86, 1.89)	2.90 (2.88, 2.93)
Magnetic resonance imaging	0.15 (0.15, 0.16)	0.38 (0.38, 0.38)	0.67 (0.66, 0.68)	1.00 (0.99, 1.01)	0.25 (0.25, 0.25)	0.58 (0.57, 0.58)	0.98 (0.98, 0.99)	1.45 (1.44, 1.47)
Radiography	3.00 (2.99, 3.02)	6.35 (6.33, 6.38)	10.17 (10.12, 10.21)	14.46 (14.39, 14.54)	3.53 (3.52, 3.55)	7.65 (7.62, 7.68)	12.49 (12.44, 12.55)	18.17 (18.08, 18.26)
Ultrasound	1.67 (1.66, 1.68)	3.45 (3.43, 3.47)	5.45 (5.43, 5.48)	7.67 (7.63, 7.71)	1.58 (1.57, 1.59)	3.35 (3.34, 3.37)	5.41 (5.38, 5.43)	7.80 (7.75, 7.84)
≥60 years at index								
Computerized tomography	0.69 (0.68, 0.70)	1.49 (1.48, 1.51)	2.48 (2.45, 2.51)	3.58 (3.53, 3.63)	1.22 (1.20, 1.23)	2.62 (2.59, 2.64)	4.39 (4.35, 4.43)	6.47 (6.40, 6.55)
Magnetic resonance imaging	0.17 (0.16, 0.17)	0.34 (0.34, 0.35)	0.54 (0.53, 0.55)	0.71 (0.69, 0.72)	0.32 (0.31, 0.32)	0.67 (0.66, 0.68)	1.06 (1.04, 1.07)	1.42 (1.39, 1.44)
Radiography	4.64 (4.61, 4.68)	8.97 (8.91, 9.03)	13.62 (13.52, 13.73)	18.10 (17.93, 18.28)	6.80 (6.76, 6.85)	14.06 (13.97, 14.14)	22.11 (21.97, 22.25)	30.65 (30.41, 30.89)
Ultrasound	1.91 (1.89, 1.92)	3.65 (3.62, 3.68)	5.46 (5.41, 5.50)	7.06 (6.99, 7.13)	2.31 (2.29, 2.33)	4.82 (4.79, 4.86)	7.58 (7.52, 7.64)	10.38 (10.28, 10.47)

95% confidence intervals provided for all estimates.
