# Supplementary material for: Medical imaging utilization in migrants compared with nonmigrants in a universal healthcare system: A population-based matched cohort study
Source: PLoS Med. 2024 Oct 22;21(10):e1004474. doi: 10.1371/journal.pmed.1004474 (PMC11495850; doi:10.1371/journal.pmed.1004474)
Supplement: S2 Table — (PDF) [file pmed.1004474.s003.pdf]

S2 Table. Number of images, rates, and measures of effect for imaging utilization of migrants and non-migrants.

Modality	Migrant				Non-migrant				Rate difference (95% CI)	Rate ratio (95% CI)
	Sample size, N	Person-years	No. of imaging exams	Incidence rate (95% CI)	Sample size, N	Person-years	No. of imaging exams	Incidence rate (95% CI)		
0-19 years at index										
	515,943	6,059,698			515,722	5,859,400				
Computerized tomography			104,702	17.3 (17.2, 17.4)			135,877	23.2 (23.1, 23.3)	-5.9 (-6.1, -5.7)	0.75 (0.74, 0.75)
Magnetic resonance imaging			80,589	13.3 (13.2, 13.4)			109,948	18.8 (18.7, 18.9)	-5.5 (-5.6, -5.3)	0.71 (0.70, 0.72)
Radiography			1,241,420	204.9 (204.5, 205.2)			1,533,041	261.6 (261.2, 262.1)	-56.8 (-57.3, -56.2)	0.78 (0.78, 0.78)
Ultrasound			899,514	148.4 (148.1, 148.7)			859,162	146.6 (146.3, 146.9)	1.8 (1.4, 2.2)	1.01 (1.01, 1.02)
20-39 years at index										
	870,219	9,725,381			870,495	9,158,592				
Computerized tomography			423,796	43.6 (43.4, 43.7)			518,653	56.6 (56.5, 56.8)	-13.1 (-13.3, -12.9)	0.77 (0.77, 0.77)
Magnetic resonance imaging			294,532	30.3 (30.2, 30.4)			397,391	43.4 (43.3, 43.5)	-13.1 (-13.3, -12.9)	0.70 (0.69, 0.70)
Radiography			3,367,098	346.2 (345.8, 346.6)			3,622,334	395.5 (395.1, 395.9)	-49.3 (-49.8, -48.7)	0.88 (0.87, 0.88)
Ultrasound			4,586,048	471.6 (471.1, 472.0)			3,810,280	416.0 (415.6, 416.5)	55.5 (54.9, 56.1)	1.13 (1.13, 1.13)
40-59 years at index										
	339,960	3,730,207			340,060	3,549,947				
Computerized tomography			327,367	87.8 (87.5, 88.1)			433,987	122.3 (121.9, 122.6)	-34.5 (-35.0, -34.0)	0.72 (0.71, 0.72)
Magnetic resonance imaging			160,495	43.0 (42.8, 43.2)			225,598	63.5 (63.3, 63.8)	-20.5 (-20.9, -20.2)	0.68 (0.67, 0.68)
Radiography			2,510,949	673.1 (672.3, 674.0)			2,917,012	821.7 (820.8, 822.6)	-148.6 (-149.8, -147.3)	0.82 (0.82, 0.82)
Ultrasound			1,351,138	362.2 (361.6, 362.8)			1,267,878	357.2 (356.5, 357.8)	5.1 (4.2, 5.9)	1.01 (1.01, 1.02)
≥60 years at index										
	123,134	1,165,843			122,979	1,002,694				
Computerized tomography			186,529	160.0 (159.3, 160.7)			279,992	279.2 (278.2, 280.3)	-119.2 (-120.5, -118.0)	0.57 (0.57, 0.58)
Magnetic resonance imaging			40,778	35.0 (34.6, 35.3)			68,387	68.2 (67.7, 68.7)	-33.2 (-33.8, -32.6)	0.51 (0.51, 0.52)
Radiography			1,064,315	912.9 (911.2, 914.6)			1,446,790	1,442.9 (1,440.6, 1,445.3)	-530.0 (-532.9, -527.1)	0.63 (0.63, 0.63)
Ultrasound			427,390	366.6 (365.5, 367.7)			493,190	491.9 (490.5, 493.2)	-125.3 (-127.0, -123.5)	0.75 (0.74, 0.75)

Rates reported per 1,000 person-years of observation. Abbreviations: 95% CI, 95% confidence interval.
