# Supplementary material for: Medical imaging utilization in migrants compared with nonmigrants in a universal healthcare system: A population-based matched cohort study
Source: PLoS Med. 2024 Oct 22;21(10):e1004474. doi: 10.1371/journal.pmed.1004474 (PMC11495850; doi:10.1371/journal.pmed.1004474)
Supplement: S3 Table — (PDF) [file pmed.1004474.s004.pdf]

S3 Table. Imaging incidence utilization by time since migration, stratified by age at migration.

S3A Table. Children and adolescents (0-19 years) at migration

Modality	Migrant				Non-migrant					
	**Sample size, N**	**Person-years**	**No. of imaging exams**	**Incidence rate (95% CI)**	**Sample size, N**	**Person-years**	**No. of imaging exams**	**Incidence rate (95% CI)**	**Rate difference (95% CI)**	**Rate ratio (95% CI)**
0 to <5 years since index										
	515,943	2,405,093			515,722	2,353,544				
Computerized tomography			26,590	11.1 (10.9, 11.2)			36,833	15.7 (15.5, 15.8)	-4.6 (-4.8, -4.4)	0.71 (0.70, 0.72)
Magnetic resonance imaging			15,803	6.6 (6.5, 6.7)			24,411	10.4 (10.2, 10.5)	-3.8 (-4.0, -3.6)	0.63 (0.62, 0.65)
Radiography			460,063	191.3 (190.7, 191.8)			590,546	250.9 (250.3, 251.6)	-59.6 (-60.5, -58.8)	0.76 (0.76, 0.77)
Ultrasound			187,364	77.9 (77.6, 78.3)			181,890	77.3 (76.9, 77.6)	0.6 (0.1, 1.1)	1.01 (1.00, 1.01)
5 to <10 years since index										
	432,709	1,863,106			421,802	1,812,772				
Computerized tomography			31,461	16.9 (16.7, 17.1)			41,040	22.6 (22.4, 22.9)	-5.8 (-6.0, -5.5)	0.75 (0.74, 0.76)
Magnetic resonance imaging			22,712	12.2 (12.0, 12.3)			32,389	17.9 (17.7, 18.1)	-5.7 (-5.9, -5.4)	0.68 (0.67, 0.69)
Radiography			384,230	206.2 (205.6, 206.9)			478,458	263.9 (263.2, 264.7)	-57.7 (-58.7, -56.7)	0.78 (0.78, 0.78)
Ultrasound			255,529	137.2 (136.6, 137.7)			238,773	131.7 (131.2, 132.2)	5.4 (4.7, 6.2)	1.04 (1.04, 1.05)
10 to <15 years since index										
	314,171	1,227,521			304,106	1,174,634				
Computerized tomography			29,267	23.8 (23.6, 24.1)			37,180	31.7 (31.3, 32.0)	-7.8 (-8.2, -7.4)	0.75 (0.74, 0.76)
Magnetic resonance imaging			25,495	20.8 (20.5, 21.0)			33,122	28.2 (27.9, 28.5)	-7.4 (-7.8, -7.0)	0.74 (0.72, 0.75)
Radiography			268,863	219.0 (218.2, 219.9)			320,836	273.1 (272.2, 274.1)	-54.1 (-55.4, -52.9)	0.80 (0.80, 0.81)
Ultrasound			272,054	221.6 (220.8, 222.5)			260,701	221.9 (221.1, 222.8)	-0.3 (-1.5, 0.9)	1.00 (0.99, 1.00)
15 to <20 years since index										
	177,792	526,557			167,354	485,409				
Computerized tomography			16,099	30.6 (30.1, 31.0)			19,276	39.7 (39.2, 40.3)	-9.1 (-9.9, -8.4)	0.77 (0.75, 0.79)
Magnetic resonance imaging			15,226	28.9 (28.5, 29.4)			18,543	38.2 (37.7, 38.8)	-9.3 (-10.0, -8.6)	0.76 (0.74, 0.77)
Radiography			119,521	227.0 (225.7, 228.3)			133,993	276.0 (274.6, 277.5)	-49.1 (-51.0, -47.1)	0.82 (0.82, 0.83)
Ultrasound			169,563	322.0 (320.5, 323.6)			163,141	336.1 (334.5, 337.7)	-14.1 (-16.3, -11.8)	0.96 (0.95, 0.96)
≥20 years since index										
	43,704	37,422			38,844	33,040				
Computerized tomography			1,338	35.8 (33.8, 37.7)			1,594	48.2 (45.9, 50.6)	-12.5 (-15.5, -9.4)	0.74 (0.69, 0.80)
Magnetic resonance imaging			1,385	37.0 (35.1, 39.0)			1,534	46.4 (44.1, 48.8)	-9.4 (-12.5, -6.4)	0.80 (0.74, 0.86)
Radiography			9,279	248.0 (242.9, 253.0)			9,793	296.4 (290.5, 302.3)	-48.4 (-56.2, -40.7)	0.84 (0.81, 0.86)
Ultrasound			15,435	412.5 (406.0, 419.0)			15,018	454.5 (447.3, 461.8)	-42.1 (-51.8, -32.3)	0.91 (0.89, 0.93)

Rates reported per 1,000 person-years of observation. Abbreviations: 95% CI, 95% confidence interval.

S3B Table. Young adults (20-39 years) at migration

Modality	Migrant				Non-migrant				Rate difference (95% CI)	Rate ratio (95% CI)
	Sample size, N	Person-years	No. of imaging exams	Incidence rate (95% CI)	Sample size, N	Person-years	No. of imaging exams	Incidence rate (95% CI)		
0 to <5 years since index										
	870,219	3,988,085			870,495	3,833,171				
Computerized tomography			129,098	32.4 (32.2, 32.5)			165,669	43.2 (43.0, 43.4)	-10.8 (-11.1, -10.6)	0.75 (0.74, 0.75)
Magnetic resonance imaging			71,293	17.9 (17.7, 18.0)			107,230	28.0 (27.8, 28.1)	-10.1 (-10.3, -9.9)	0.64 (0.63, 0.65)
Radiography			1,174,748	294.6 (294.0, 295.1)			1,241,074	323.8 (323.2, 324.3)	-29.2 (-30.0, -28.4)	0.91 (0.91, 0.91)
Ultrasound			2,001,161	501.8 (501.1, 502.5)			1,591,368	415.2 (414.5, 415.8)	86.6 (85.7, 87.6)	1.21 (1.21, 1.21)
5 to <10 years since index										
	701,021	2,980,820			664,844	2,813,470				
Computerized tomography			129,635	43.5 (43.3, 43.7)			156,156	55.5 (55.2, 55.8)	-12.0 (-12.4, -11.7)	0.78 (0.78, 0.79)
Magnetic resonance imaging			90,013	30.2 (30.0, 30.4)			121,999	43.4 (43.1, 43.6)	-13.2 (-13.5, -12.9)	0.70 (0.69, 0.70)
Radiography			1,004,761	337.1 (336.4, 337.7)			1,076,108	382.5 (381.8, 383.2)	-45.4 (-46.4, -44.4)	0.88 (0.88, 0.88)
Ultrasound			1,417,488	475.5 (474.8, 476.3)			1,225,368	435.5 (434.8, 436.3)	40.0 (38.9, 41.1)	1.09 (1.09, 1.09)
10 to <15 years since index										
	492,468	1,913,893			461,022	1,767,145				
Computerized tomography			106,528	55.7 (55.3, 56.0)			127,346	72.1 (71.7, 72.5)	-16.4 (-16.9, -15.9)	0.77 (0.77, 0.78)
Magnetic resonance imaging			85,790	44.8 (44.5, 45.1)			109,754	62.1 (61.7, 62.5)	-17.3 (-17.8, -16.8)	0.72 (0.72, 0.73)
Radiography			774,016	404.4 (403.5, 405.3)			855,856	484.3 (483.3, 485.3)	-79.9 (-81.3, -78.5)	0.84 (0.83, 0.84)
Ultrasound			820,321	428.6 (427.7, 429.5)			708,768	401.1 (400.1, 402.0)	27.5 (26.2, 28.8)	1.07 (1.07, 1.07)
15 to <20 years since index										
	272,827	789,303			247,386	699,566				
Computerized tomography			54,306	68.8 (68.2, 69.4)			64,441	92.1 (91.4, 92.8)	-23.3 (-24.2, -22.4)	0.75 (0.74, 0.76)
Magnetic resonance imaging			44,201	56.0 (55.5, 56.5)			54,628	78.1 (77.4, 78.7)	-22.1 (-22.9, -21.3)	0.72 (0.71, 0.73)
Radiography			384,555	487.2 (485.7, 488.7)			418,722	598.5 (596.7, 600.4)	-111.3 (-113.7, -109.0)	0.81 (0.81, 0.82)
Ultrasound			325,557	412.5 (411.0, 413.9)			267,839	382.9 (381.4, 384.3)	29.6 (27.6, 31.6)	1.08 (1.07, 1.08)
≥20 years since index										
	62,152	53,281			52,921	45,241				
Computerized tomography			4,421	83.0 (80.5, 85.4)			5,255	116.2 (113.0, 119.3)	-33.2 (-37.2, -29.2)	0.71 (0.69, 0.74)
Magnetic resonance imaging			3,392	63.7 (61.5, 65.8)			3,940	87.1 (84.4, 89.8)	-23.4 (-26.9, -20.0)	0.73 (0.70, 0.77)
Radiography			30,482	572.1 (565.7, 578.5)			32,133	710.3 (702.5, 718.0)	-138.2 (-148.2, -128.1)	0.81 (0.79, 0.82)
Ultrasound			23,386	438.9 (433.3, 444.5)			18,601	411.2 (405.2, 417.1)	27.8 (19.6, 35.9)	1.07 (1.05, 1.09)

Rates reported per 1,000 person-years of observation. Abbreviations: 95% CI, 95% confidence interval.

S3C Table. Adults (40-59 years) at migration

Modality	Migrant				Non-migrant					
	**Sample size, N**	**Person-years**	**No. of imaging exams**	**Incidence rate (95% CI)**	**Sample size, N**	**Person-years**	**No. of imaging exams**	**Incidence rate (95% CI)**	**Rate difference (95% CI)**	**Rate ratio (95% CI)**
0 to <5 years since index										
	339,960	1,557,880			340,060	1,517,268				
Computerized tomography			101,329	65.0 (64.6, 65.4)			142,019	93.6 (93.1, 94.1)	-28.6 (-29.2, -27.9)	0.69 (0.69, 0.70)
Magnetic resonance imaging			47,629	30.6 (30.3, 30.8)			74,971	49.4 (49.1, 49.8)	-18.8 (-19.3, -18.4)	0.62 (0.61, 0.63)
Radiography			936,676	601.3 (600.0, 602.5)			1,071,012	705.9 (704.5, 707.2)	-104.6 (-106.4, -102.8)	0.85 (0.85, 0.85)
Ultrasound			521,598	334.8 (333.9, 335.7)			481,414	317.3 (316.4, 318.2)	17.5 (16.2, 18.8)	1.06 (1.05, 1.06)
5 to <10 years since index										
	273,591	1,147,664			263,306	1,095,521				
Computerized tomography			99,899	87.0 (86.5, 87.6)			131,225	119.8 (119.1, 120.4)	-32.7 (-33.6, -31.9)	0.73 (0.72, 0.73)
Magnetic resonance imaging			51,390	44.8 (44.4, 45.2)			71,068	64.9 (64.4, 65.3)	-20.1 (-20.7, -19.5)	0.69 (0.68, 0.70)
Radiography			766,402	667.8 (666.3, 669.3)			897,897	819.6 (817.9, 821.3)	-151.8 (-154.1, -149.6)	0.81 (0.81, 0.82)
Ultrasound			408,040	355.5 (354.4, 356.6)			386,230	352.6 (351.4, 353.7)	3.0 (1.4, 4.5)	1.01 (1.00, 1.01)
10 to <15 years since index										
	186,374	706,743			175,765	655,255				
Computerized tomography			80,304	113.6 (112.8, 114.4)			103,432	157.9 (156.9, 158.8)	-44.2 (-45.5, -43.0)	0.72 (0.71, 0.73)
Magnetic resonance imaging			40,703	57.6 (57.0, 58.2)			53,327	81.4 (80.7, 82.1)	-23.8 (-24.7, -22.9)	0.71 (0.70, 0.72)
Radiography			536,944	759.7 (757.7, 761.8)			631,649	964.0 (961.6, 966.4)	-204.2 (-207.4, -201.1)	0.79 (0.79, 0.79)
Ultrasound			281,890	398.9 (397.4, 400.3)			267,439	408.1 (406.6, 409.7)	-9.3 (-11.4, -7.2)	0.98 (0.97, 0.98)
15 to <20 years since index										
	99,362	296,854			90,182	263,869				
Computerized tomography			42,406	142.9 (141.5, 144.2)			52,979	200.8 (199.1, 202.5)	-57.9 (-60.1, -55.7)	0.71 (0.70, 0.72)
Magnetic resonance imaging			19,415	65.4 (64.5, 66.3)			24,527	93.0 (91.8, 94.1)	-27.5 (-29.0, -26.1)	0.70 (0.69, 0.72)
Radiography			252,551	850.8 (847.4, 854.1)			295,078	1,118.3 (1,114.2, 1,122.3)	-267.5 (-272.7, -262.3)	0.76 (0.76, 0.76)
Ultrasound			130,046	438.1 (435.7, 440.5)			123,728	468.9 (466.3, 471.5)	-30.8 (-34.4, -27.3)	0.93 (0.93, 0.94)
≥20 years since index										
	24,978	21,067			21,444	18,034				
Computerized tomography			3,586	170.2 (164.6, 175.8)			4,515	250.4 (243.1, 257.7)	-80.1 (-89.3, -71.0)	0.68 (0.65, 0.71)
Magnetic resonance imaging			1,427	67.7 (64.2, 71.3)			1,798	99.7 (95.1, 104.3)	-32.0 (-37.8, -26.2)	0.68 (0.63, 0.73)
Radiography			19,441	922.8 (909.9, 935.8)			22,591	1,252.7 (1,236.3, 1,269.0)	-329.9 (-350.7, -309.0)	0.74 (0.72, 0.75)
Ultrasound			10,099	479.4 (470.0, 488.7)			9,627	533.8 (523.2, 544.5)	-54.4 (-68.6, -40.3)	0.90 (0.87, 0.92)

Rates reported per 1,000 person-years of observation. Abbreviations: 95% CI, 95% confidence interval.

S3D Table. Older adults (≥60 years) at migration

Modality	Migrant				Non-migrant				Rate difference (95% CI)	Rate ratio (95% CI)
	Sample size, N	Person-years	No. of imaging exams	Incidence rate (95% CI)	Sample size, N	Person-years	No. of imaging exams	Incidence rate (95% CI)		
0 to <5 years since index										
	123,134	540,413			122,979	502,822				
Computerized tomography			74,572	138.0 (137.0, 139.0)			122,770	244.2 (242.8, 245.5)	-106.2 (-107.9, -104.5)	0.57 (0.56, 0.57)
Magnetic resonance imaging			17,947	33.2 (32.7, 33.7)			32,115	63.9 (63.2, 64.6)	-30.7 (-31.5, -29.8)	0.52 (0.51, 0.53)
Radiography			508,021	940.1 (937.5, 942.6)			685,003	1,362.3 (1,359.1, 1,365.5)	-422.3 (-426.4, -418.1)	0.69 (0.69, 0.69)
Ultrasound			208,967	386.7 (385.0, 388.3)			232,162	461.7 (459.8, 463.6)	-75.0 (-77.5, -72.5)	0.84 (0.83, 0.84)
5 to <10 years since index										
	85,315	343,819			74,939	291,039				
Computerized tomography			54,930	159.8 (158.4, 161.1)			80,654	277.1 (275.2, 279.0)	-117.4 (-119.7, -115.0)	0.58 (0.57, 0.58)
Magnetic resonance imaging			12,286	35.7 (35.1, 36.4)			20,504	70.5 (69.5, 71.4)	-34.7 (-35.9, -33.6)	0.51 (0.50, 0.52)
Radiography			297,161	864.3 (861.2, 867.4)			421,172	1,447.1 (1,442.8, 1,451.5)	-582.8 (-588.2, -577.5)	0.60 (0.59, 0.60)
Ultrasound			119,729	348.2 (346.3, 350.2)			145,677	500.5 (498.0, 503.1)	-152.3 (-155.5, -149.1)	0.70 (0.69, 0.70)
10 to <15 years since index										
	52,928	197,884			42,827	152,696				
Computerized tomography			38,877	196.5 (194.5, 198.4)			53,492	350.3 (347.3, 353.3)	-153.9 (-157.4, -150.3)	0.56 (0.55, 0.57)
Magnetic resonance imaging			7,667	38.7 (37.9, 39.6)			11,705	76.7 (75.3, 78.0)	-37.9 (-39.5, -36.3)	0.51 (0.49, 0.52)
Radiography			184,433	932.0 (927.8, 936.3)			245,444	1,607.4 (1,601.0, 1,613.8)	-675.4 (-683.0, -667.7)	0.58 (0.58, 0.58)
Ultrasound			71,890	363.3 (360.6, 365.9)			83,933	549.7 (546.0, 553.4)	-186.4 (-191.0, -181.8)	0.66 (0.65, 0.67)
15 to <20 years since index										
	26,062	77,645			18,824	52,511				
Computerized tomography			16,903	217.7 (214.4, 221.0)			21,368	406.9 (401.5, 412.4)	-189.2 (-195.6, -182.9)	0.53 (0.52, 0.55)
Magnetic resonance imaging			2,717	35.0 (33.7, 36.3)			3,819	72.7 (70.4, 75.0)	-37.7 (-40.4, -35.1)	0.48 (0.46, 0.51)
Radiography			70,171	903.7 (897.1, 910.4)			89,019	1,695.2 (1,684.1, 1,706.4)	-791.5 (-804.5, -778.5)	0.53 (0.53, 0.54)
Ultrasound			25,339	326.3 (322.3, 330.4)			29,471	561.2 (554.8, 567.6)	-234.9 (-242.5, -227.3)	0.58 (0.57, 0.59)
≥20 years since index										
	6,968	6,082			4,229	3,626				
Computerized tomography			1,326	218.0 (206.3, 229.8)			1,816	500.8 (477.8, 523.8)	-282.8 (-308.6, -256.9)	0.44 (0.41, 0.47)
Magnetic resonance imaging			191	31.4 (27.0, 35.9)			272	75.0 (66.1, 83.9)	-43.6 (-53.6, -33.6)	0.42 (0.35, 0.50)
Radiography			4,925	809.8 (787.2, 832.4)			6,730	1,855.9 (1,811.6, 1,900.3)	-1,046.1 (-1,095.9, -996.4)	0.44 (0.42, 0.45)
Ultrasound			1,616	265.7 (252.8, 278.7)			2,143	591.0 (566.0, 616.0)	-325.3 (-353.4, -297.1)	0.45 (0.42, 0.48)

Rates reported per 1,000 person-years of observation. Abbreviations: 95% CI, 95% confidence interval.
