# Supplementary material for: Medical imaging utilization in migrants compared with nonmigrants in a universal healthcare system: A population-based matched cohort study
Source: PLoS Med. 2024 Oct 22;21(10):e1004474. doi: 10.1371/journal.pmed.1004474 (PMC11495850; doi:10.1371/journal.pmed.1004474)
Supplement: S4 Table — (PDF) [file pmed.1004474.s005.pdf]

S4 Table. Sex-specific relative rate of medical imaging utilization stratified by age at migration.

Age at index	Relative rate (95% CI)			
	**Computerized tomography**	**Magnetic resonance imaging**	**Radiography**	**Ultrasound**
Male				
0-19 years	0.81 (0.80, 0.81)	0.84 (0.82, 0.85)	0.88 (0.87, 0.88)	1.07 (1.07, 1.08)
20-39 years	0.76 (0.76, 0.77)	0.75 (0.75, 0.76)	0.86 (0.86, 0.86)	1.19 (1.19, 1.20)
40-59 years	0.75 (0.75, 0.76)	0.78 (0.77, 0.79)	0.86 (0.86, 0.87)	1.08 (1.08, 1.09)
$\geq$60 years	0.73 (0.72, 0.74)	0.73 (0.72, 0.75)	0.83 (0.83, 0.84)	0.95 (0.94, 0.95)
Female				
0-19 years	0.80 (0.79, 0.80)	0.76 (0.75, 0.77)	0.84 (0.84, 0.84)	1.09 (1.08, 1.09)
20-39 years	0.78 (0.78, 0.79)	0.73 (0.73, 0.73)	0.91 (0.91, 0.91)	1.21 (1.21, 1.21)
40-59 years	0.83 (0.82, 0.83)	0.79 (0.79, 0.80)	0.91 (0.91, 0.92)	1.14 (1.14, 1.15)
$\geq$60 years	0.81 (0.81, 0.82)	0.71 (0.70, 0.73)	0.84 (0.84, 0.84)	1.00 (1.00, 1.01)

Models are stratified by age at index and sex. Results represent the relative rate of imaging for migrants compared with the reference group of matched non-migrants. Models are adjusted for age, migration year, and time-varying socioeconomic status, Aggregated Diagnostic Group score, and visits to a primary care provider. Time-varying covariates were updated annually until the end of observation. Abbreviations: 95%CI, 95% confidence interval.
