# Supplementary material for: Medical imaging utilization in migrants compared with nonmigrants in a universal healthcare system: A population-based matched cohort study
Source: PLoS Med. 2024 Oct 22;21(10):e1004474. doi: 10.1371/journal.pmed.1004474 (PMC11495850; doi:10.1371/journal.pmed.1004474)
Supplement: S5 Table — (PDF) [file pmed.1004474.s006.pdf]

S5 Table. Relative rate of medical imaging utilization stratified by age at migration and migration year.

		Relative rate (95% CI)							
		Male				Female			
Age at index	Migration year	Computerized tomography	Magnetic resonance imaging	Radiography	Ultrasound	Computerized tomography	Magnetic resonance imaging	Radiography	Ultrasound
0-19 years	1995-1999	0.83 (0.82, 0.85)	0.83 (0.81, 0.85)	0.89 (0.88, 0.89)	1.05 (1.04, 1.06)*	0.78 (0.77, 0.80)	0.75 (0.73, 0.77)	0.83 (0.83, 0.84)	1.06 (1.06, 1.07)*
	2000-2004	0.81 (0.80, 0.83)	0.85 (0.83, 0.87)	0.87 (0.86, 0.87)	1.04 (1.03, 1.05)	0.76 (0.75, 0.78)	0.76 (0.74, 0.78)	0.82 (0.82, 0.83)	1.10 (1.09, 1.11)
	2005-2009	0.78 (0.76, 0.80)*	0.81 (0.79, 0.84)	0.89 (0.88, 0.89)	1.06 (1.04, 1.07)	0.80 (0.78, 0.82)*	0.77 (0.74, 0.79)	0.83 (0.82, 0.84)	1.15 (1.14, 1.16)
	2010-2016	0.78 (0.74, 0.82)*	0.86 (0.82, 0.91)	0.91 (0.90, 0.92)	1.11 (1.09, 1.14)*	0.76 (0.71, 0.80)*	0.75 (0.71, 0.79)	0.84 (0.83, 0.85)	1.09 (1.08, 1.11)*
20-39 years	1995-1999	0.74 (0.74, 0.75)	0.71 (0.70, 0.72)	0.85 (0.85, 0.86)	1.20 (1.20, 1.21)	0.79 (0.79, 0.80)	0.76 (0.75, 0.77)	0.91 (0.91, 0.92)	1.16 (1.16, 1.16)
	2000-2004	0.74 (0.73, 0.75)	0.71 (0.70, 0.72)*	0.84 (0.84, 0.84)	1.21 (1.20, 1.22)	0.78 (0.78, 0.79)	0.72 (0.71, 0.72)*	0.91 (0.91, 0.91)	1.16 (1.16, 1.16)
	2005-2009	0.79 (0.78, 0.80)	0.79 (0.78, 0.80)	0.89 (0.88, 0.89)	1.27 (1.26, 1.28)	0.76 (0.75, 0.77)	0.72 (0.71, 0.73)	0.89 (0.89, 0.90)	1.22 (1.22, 1.22)
	2010-2016	0.79 (0.77, 0.80)	0.84 (0.82, 0.86)	0.90 (0.89, 0.91)	1.30 (1.28, 1.32)	0.73 (0.71, 0.74)	0.68 (0.67, 0.70)	0.86 (0.85, 0.87)	1.32 (1.31, 1.32)
40-59 years	1995-1999	0.77 (0.77, 0.78)	0.76 (0.75, 0.78)	0.88 (0.88, 0.89)	1.05 (1.05, 1.06)	0.82 (0.81, 0.83)	0.78 (0.77, 0.80)	0.90 (0.89, 0.90)	1.10 (1.09, 1.11)
	2000-2004	0.74 (0.73, 0.75)	0.75 (0.74, 0.77)	0.85 (0.85, 0.85)	1.08 (1.07, 1.08)	0.84 (0.83, 0.85)	0.78 (0.77, 0.79)	0.91 (0.91, 0.92)	1.12 (1.12, 1.13)
	2005-2009	0.73 (0.72, 0.74)	0.77 (0.75, 0.78)*	0.85 (0.84, 0.85)	1.12 (1.11, 1.13)	0.82 (0.81, 0.83)	0.78 (0.77, 0.80)*	0.94 (0.93, 0.94)	1.16 (1.15, 1.16)
	2010-2016	0.74 (0.72, 0.75)	0.82 (0.80, 0.84)	0.87 (0.86, 0.88)	1.16 (1.14, 1.17)	0.80 (0.78, 0.81)	0.78 (0.76, 0.80)	0.94 (0.93, 0.94)	1.20 (1.19, 1.21)
≥60 years	1995-1999	0.74 (0.73, 0.76)	0.75 (0.72, 0.79)	0.84 (0.83, 0.85)*	0.92 (0.91, 0.94)	0.82 (0.81, 0.83)	0.66 (0.64, 0.69)	0.84 (0.84, 0.85)*	0.97 (0.96, 0.98)
	2000-2004	0.72 (0.71, 0.74)	0.71 (0.68, 0.73)*	0.82 (0.81, 0.83)	0.94 (0.93, 0.95)	0.82 (0.81, 0.83)	0.69 (0.67, 0.71)*	0.85 (0.84, 0.85)	1.00 (0.99, 1.01)
	2005-2009	0.69 (0.68, 0.71)	0.71 (0.68, 0.73)*	0.78 (0.78, 0.79)	0.92 (0.90, 0.93)	0.77 (0.76, 0.78)	0.68 (0.66, 0.71)*	0.82 (0.82, 0.83)	0.97 (0.96, 0.98)
	2010-2016	0.72 (0.70, 0.74)	0.75 (0.72, 0.78)*	0.81 (0.80, 0.82)	1.00 (0.98, 1.01)	0.81 (0.79, 0.82)	0.76 (0.73, 0.78)*	0.87 (0.86, 0.87)	1.06 (1.04, 1.07)

Models are stratified by age at index and migration year. Results represent the relative rate of imaging for migrants compared with the reference group of matched non-migrants. Models contain an interaction between migration status and sex. Adjusted for age, time-varying socioeconomic status, Aggregated Diagnostic Group score, and visits to a primary care provider. Time-varying covariates were updated annually until the end of observation. Abbreviations: 95%CI, 95% confidence interval.
* P-value for migrant status & sex interaction > 0.05.
