# Supplementary material for: Medical imaging utilization in migrants compared with nonmigrants in a universal healthcare system: A population-based matched cohort study
Source: PLoS Med. 2024 Oct 22;21(10):e1004474. doi: 10.1371/journal.pmed.1004474 (PMC11495850; doi:10.1371/journal.pmed.1004474)
Supplement: S6 Table — (PDF) [file pmed.1004474.s007.pdf]

S6 Table. Relative rate of medical imaging utilization stratified by age at migration using an index date 2-years later.

Age at index	Relative rate (95% CI)			
	**Computerized tomography**	**Magnetic resonance imaging**	**Radiography**	**Ultrasound**
0-19 years	0.80 (0.79, 0.80)	0.79 (0.78, 0.80)	0.85 (0.84, 0.85)	1.07 (1.06, 1.07)
20-39 years	0.77 (0.77, 0.78)	0.74 (0.73, 0.74)	0.87 (0.87, 0.87)	1.14 (1.14, 1.14)
40-59 years	0.80 (0.79, 0.80)	0.78 (0.78, 0.79)	0.87 (0.87, 0.87)	1.08 (1.08, 1.08)
$\geq$60 years	0.76 (0.75, 0.76)	0.69 (0.68, 0.70)	0.79 (0.79, 0.79)	0.91 (0.90, 0.91)

Age-stratified models represent the relative rate of imaging for migrants compared with the reference group of matched non-migrants. Models are adjusted for age, sex, migration year, and time-varying socioeconomic status, Aggregated Diagnostic Group score, and visits to a primary care provider. Time-varying covariates were updated annually until the end of observation. Abbreviations: 95%CI, 95% confidence interval.
