# Supplementary material for: Medical imaging utilization in migrants compared with nonmigrants in a universal healthcare system: A population-based matched cohort study
Source: PLoS Med. 2024 Oct 22;21(10):e1004474. doi: 10.1371/journal.pmed.1004474 (PMC11495850; doi:10.1371/journal.pmed.1004474)

S1 Fig. Average comorbidity score during the first 10 years of follow-up for migrant and non-migrants

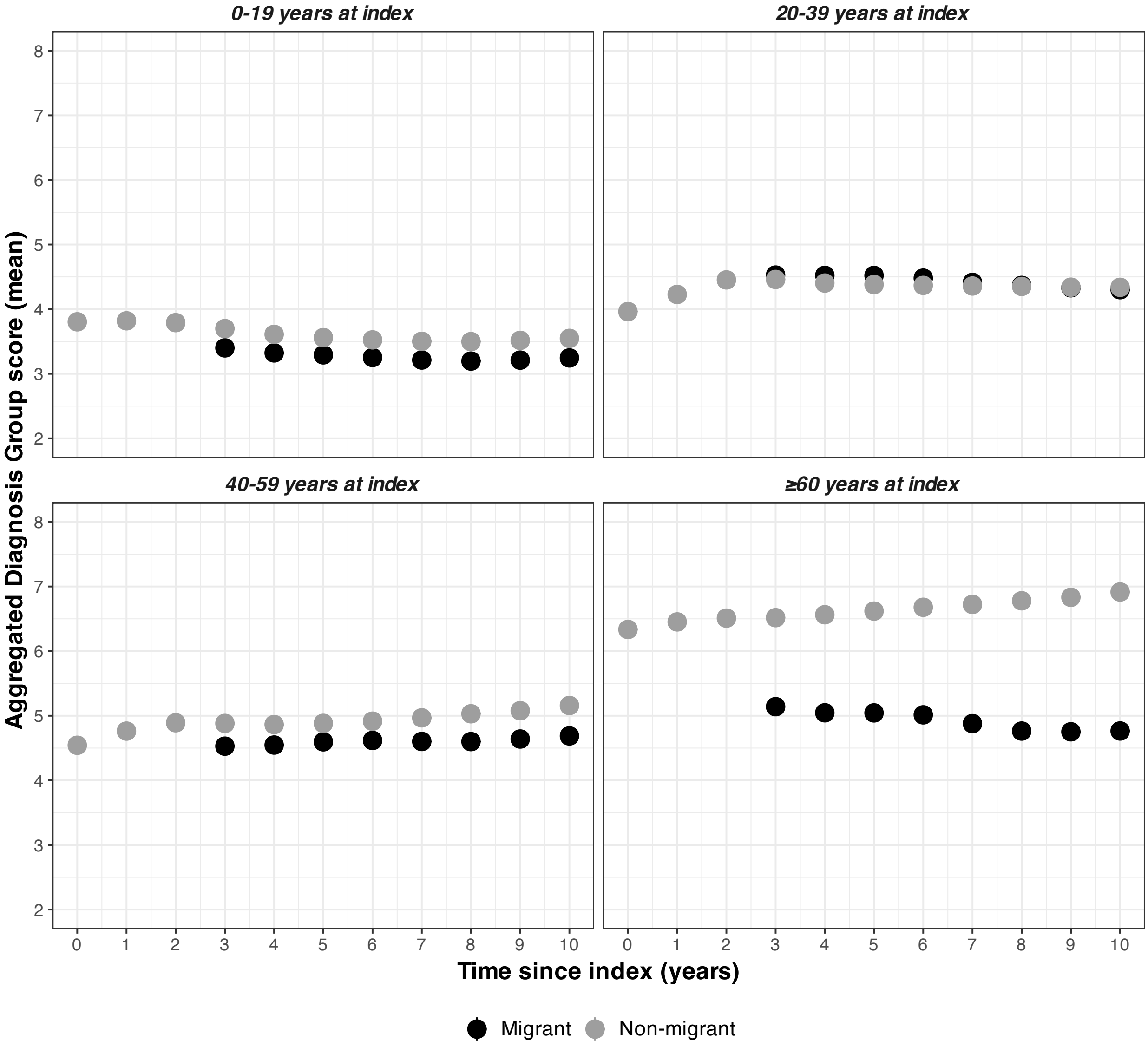

Supplement: S1 Fig — (PDF) [file pmed.1004474.s008.pdf]
