# Supplementary material for: Medical imaging utilization in migrants compared with nonmigrants in a universal healthcare system: A population-based matched cohort study
Source: PLoS Med. 2024 Oct 22;21(10):e1004474. doi: 10.1371/journal.pmed.1004474 (PMC11495850; doi:10.1371/journal.pmed.1004474)

S2 Fig. Utilization of head computerized tomography within 7-days of an emergency department visit for a head trauma.

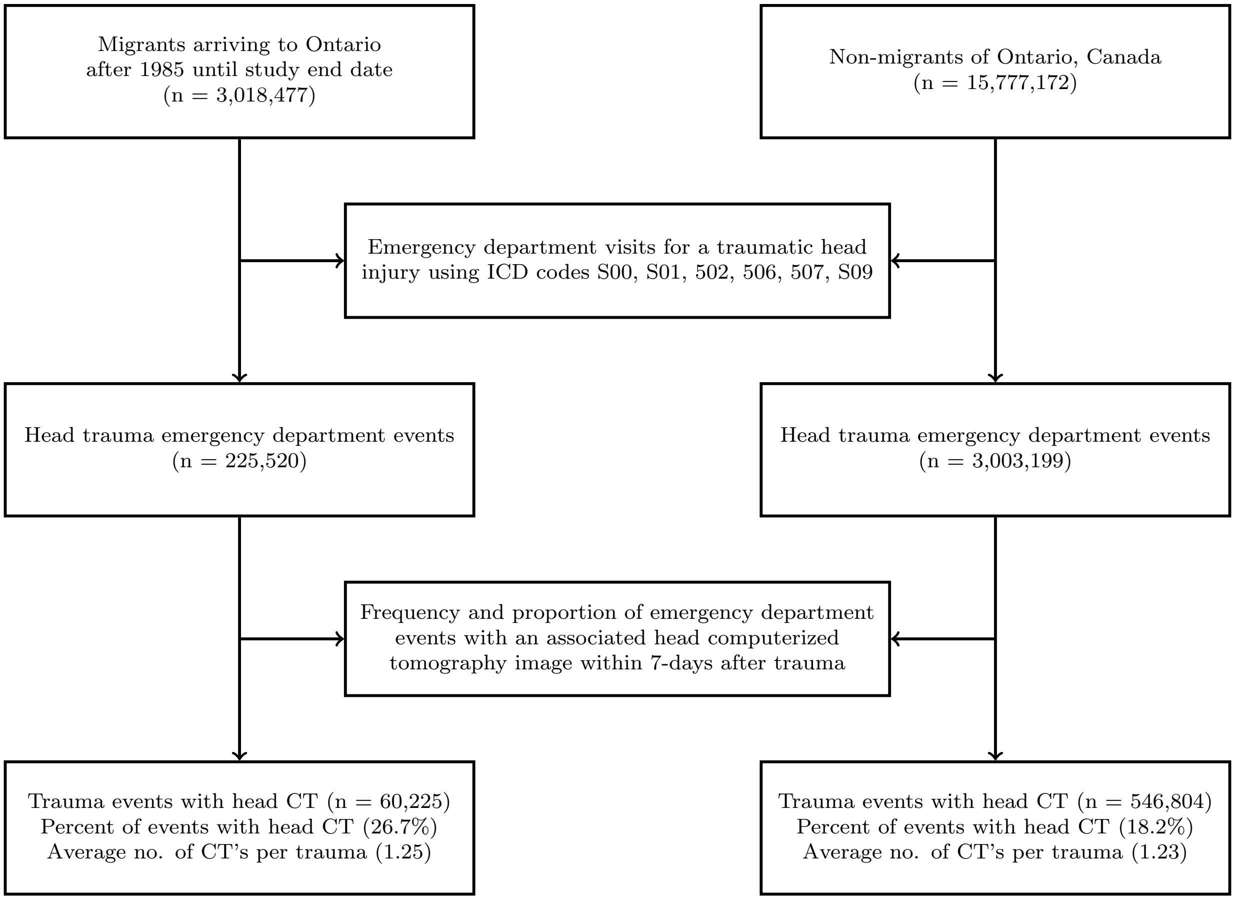

Supplement: S2 Fig — (PDF) [file pmed.1004474.s009.pdf]
