# Supplementary material for: Medical imaging utilization in migrants compared with nonmigrants in a universal healthcare system: A population-based matched cohort study
Source: PLoS Med. 2024 Oct 22;21(10):e1004474. doi: 10.1371/journal.pmed.1004474 (PMC11495850; doi:10.1371/journal.pmed.1004474)
Supplement: Attachment — Submitted filename: Response_to_reviewers R4.pdf [file pmed.1004474.s013.pdf]

Comments from the Academic Editor and editorial Team

Thank you very much for your revised manuscript. We find it much improved and only have minor requests at this stage:

1) The Academic Editor is still not entirely convinced by your response to the interaction comment. We propose that you are explicit, and consider changing the sentence to:

"This suggests a need for future research to explore other mechanisms which may aid in explaining the lowered utilization of medical imaging or if there is heterogeneity in the effects of being a migrant on utilization across factors are effect modifiers such having a PCP or income."

> **Response:** Thank you for continuing to push for clarity on this issue. We have accepted your suggested wording and edited the appropriate Discussion section.

2) In the 'Abstract Methods and Findings' section, in the last sentence of the Abstract Methods and Findings section, please describe the main limitation(s) of the study's methodology.

> **Response:** The main limitation as noted is not having information pertaining to indication for the exam. We have now included this as the last sentence in the abstract as requested.

Comments from the reviewers:

> **Response:** The comments from reviewers included were addressed in the previous round of revisions.
