# Supplementary material for: Medical imaging utilization in migrants compared with nonmigrants in a universal healthcare system: A population-based matched cohort study
Source: PLoS Med. 2024 Oct 22;21(10):e1004474. doi: 10.1371/journal.pmed.1004474 (PMC11495850; doi:10.1371/journal.pmed.1004474)
Supplement: Attachment — Submitted filename: Peer Review PLOS Medicine.docx [file pmed.1004474.s010.docx]

Reviewer’s Report –

**Immigrants medical imaging utilization compared to residents in a universal healthcare system**

**Review for PLOS Medicine**

**Manuscript number:** PMEDICINE-D-24-00454R1

Summary: This paper presents an empirical investigation as to how the usage of medical imaging healthcare by ‘immigrants’ in the state of Ontario, Canada, compares to residents. The work benefits from a rich administrative data set – with 21 years of data 1995-2016 of complete records for CT, MRI, radiography, and ultrasonography utilisation of patients – with important patient information including information on gender, age, comorbidity which allows for controlling of key explanatory variables. The statistical methodology is appropriate and well-executed – ways of backfilling data etc to deal with data gaps are appropriate, the sensitivity analysis with CT utilisation for traumatic brain injury is very nice. The Tables and charts are well presented. I have some concerns about how some aspects of the paper are described – and have additional suggestions to improve the contextual understanding and presentation of the work for an international readership – outlined in this peer- review.

Overall, I see the merit in the research, and recommend revisions of the content and presentation of the work undertaken which where address can allow for the paper to progress towards publication in *PLOS Medicine*.

Title/Abstract – Throughout this paper (and even the labelling on your charts!) I do not agree with the distinction between the groups as immigrants versus residents of Canada. The immigrant groups do in fact appear to be residents – so surely it is more accurate to describe the groups as native versus non-native residents of Canada – from what I gather they are distinguished by country of birth i.e. nationality.

The reference to matching 1 to 1 on residents based on age, sex, **immigration year (?)**, and geography is confusing – how can you match native residents versus immigrant residents based on immigration year – this does not make sense to me at least on first read? Please clarify if this is a typo or something else?

The results accord with expectations apart from the result on the longer the time since immigration the larger the gap – interesting and slightly counterintuitive.

Keywords- I don’t think the use of emigrants under this paper is appropriate – also I do not think this is a traditional cohort study – so this terminology is misleading (?)

Introduction - please provide clarification on your resident and migrant distinction.

Since Canada is a universal healthcare system in which GPs are the main gatekeepers – and GPs can be a key route through which patients access medical imaging, it is important to also cite literature which examines immigrants access to GP services – e.g. Barlow, Mohan, and Nolan (2022) Utilisation of healthcare by immigrant adults relative to the host population: Evidence from Ireland. *Journal of Migration and Health*.

Line 85: radiology **use or access**

Line 87: crucial to gain insight into any disparities in immigrant populations for **policy making and healthcare planning**… Please refer to important policy documents from the WHO in this area e.g. World Health Organization, 2019. Promoting the Health of Refugees and Migrants Draft Global Action Plan, 2019–2023. <https://apps.who.int/gb/ebwha/pdf_files/WHA72/A72_25-en.pdf>.

It would also be good to know if the Canadian government or a Canadian Medical Association – or locally in Ontario had policies published on migrant health – there is no enough discussion of policies and planning in this area – particularly if there is any consideration of this in terms of radiology which is a unique selling point of your paper – you do not sufficiently sell the special case of native v non-natives use of medical imaging as a motivation for your research – the use or non-use of this aspect of healthcare obviously has downstream effects e.g. lack of detection/diagnosis leads to poorer patient outcomes (more advanced cancers etc), preventable mortality etc . – provide references to strengthen your arguments. Greater sell the novelty and wider importance of this work.

The work also does not provide much consideration of the world beyond the Canadian context – how generalisable are the findings? What lessons can be gleemed for other countries/regions and jurisdictions?

You do not provide a clear research question – no outline a clear aim of the research. The hypothesis you provide is not supported by any other empirical references or a previous theoretical underpinning – this is needed to strengthen your argument. – Is it an assimilation or integration argument that you are making – the longer a person is resident in a country they adopt the norms of the local population, including healthcare utilisation norms?

And then in testing this hypothesis I see later you have categories of >10 years in Canada, > 20 years etc – is the hypothesis based on any particular cut off period (e.g. over 10/20 years) or just a gradient over time should be seen?

Methods

You have really comprehensive coverage on migrants. I am uncomfortable with the use of the term ‘landed’ throughout – maybe arrival/taking up residency is a better way of phrasing this?

It is not clear whether or not you have the exact country or region of origin of migrants in your dataset – if not, please clarify (maybe I have missed this) – the literature has shown that there can be significantly heterogenous findings on healthcare utilisation by different migrant groups – and you are treating them all as a homogenous group. For example, Mohan (2021) The influence of caregiver's migration status on child's use of healthcare services: evidence from Ireland, *Sociology of Health and Illness,* finds different patterns in use of healthcare services by children of different migrant groups e.g. those from less advanced non-English speaking non-European countries availed less of GP services less than native population – and other types of migrant groups. This paper may also be relevant for commentary on your 0-19 year old group – since children (of migrant background) rely on their parents/caregivers to help them access (and are involved in the decision making process around) healthcare services – and the parents are likely to be of a migrant background themselves.

You may need to better describe in the Introduction the typical pathways for a patient to access medical imaging services. For example, describing the GP as gatekeeper role – which I assume is free at the point of use (or is there any co-payments) and then if there are co-payments in services – the reason I query this here is you say that a source of your medical imaging data is from fee-for-service physician billing records (OHIP Schedule of Benefits and Fees) – and an international readership will not understand what this is under a universal healthcare system. In the UK’s NHS which is a universal healthcare system there are no fees for services – e.g. copayments – so outlining the institutional setting is important.

Please define meaning of ICES.

Is your control variable of a visit to a primary care provider a binary variable – and is that in the prior 12-month period of what is the exact definition?

Discussion

Your surprising result which is nice that you can test in the data that you have (many studies do not have duration of residence in the country) – was that the longer a person is resident in Canda there medical imaging use remained lower than that of their Canadian counterparts – more could be commented on this again bringing in theory or supporting references on lack of assimilation/integration. The point you make that ‘passively waiting for access for new immigrants to improve over time does not occur’ is a key indication from this paper – to which more could be made of this in terms of evidence gathering to inform national government policies and the like of supranational organisations such as the WHO, UN, EU etc.

The point on older migrant groups accessing services less is also insightful – you do not have data on whether they have children or not – but again the family circumstances of the household are likely to be key here (as in the case of children previously mentioned) – so while you can’t answer this question it may be that older immigrant people with children may be better able to access services if there is a language barrier etc. – as the children can speak on their behalf – i.e. there may even be heterogenous access within this group depending on family circumstances.

A reason for lower utilisation of services by immigrant groups which has not been mentioned is that sometime immigrant groups go back to their home country of origin of care e.g. Wallace et al (2009) showed that Mexican migrants in the US went home to Mexico to receive care – this may not be to the same extent in the Canadian setting.

Again, the policy and practice implications and the relevance for an international audience needed greater consideration.

Overall, great data and well executed analysis which could be valuable where the paper is strengthened.
