# Supplementary material for: Medical imaging utilization in migrants compared with nonmigrants in a universal healthcare system: A population-based matched cohort study
Source: PLoS Med. 2024 Oct 22;21(10):e1004474. doi: 10.1371/journal.pmed.1004474 (PMC11495850; doi:10.1371/journal.pmed.1004474)
Supplement: Attachment — Submitted filename: Review_feedback_R2_PLoS Med.docx [file pmed.1004474.s011.docx]

**EDITORIAL COMMENTS:**

From an editorial perspective, we feel that the study is a bit superficial at the moment and that we feel there is potential for a much richer analysis, along the lines as suggestions by Reviewer 3. The Academic Editor also mentioned the following possibility "stratifying by having a PCP vs. not (there was a HUGE difference in this), index time (have things improved over secular time?), income quantile, geography, diagnosis codes, etc. rather than adjusting for all these, as stratifying and identifying interaction could have been somewhat more illuminating and may have spoken to underlying drivers a bit more." It is not necessary that you have to go about in that manner, but it is an option for helping to deepen the manuscript which we do feel is required.

**Response:** Two additional analyses were performed based on feedback from the editorial team and reviewers.

First, we further stratified our multivariable regression models by sex (in addition to age at immigration) to uncover potential sex differences in medical imaging utilization. This has been added as S4 Table in the supplement. Secondly, to explore if any changes over time have occurred with the secular time of immigration, we have conducted an analysis whereby we stratify all multivariable regression models by age at index and the year of immigration. These models also contain an interaction with sex given the feedback provided by Reviewer #4. This is now provided in S5 Table.

We have added these analyses to the methods and results:

“Age- and sex-stratified multivariable regression models were performed *ad hoc* to explore the effect of sex on the relative rate of imaging. Additionally, an ad hoc analysis was performed by stratifying the regression models by age at index and the grouped year of immigration (1995-1999, 2000-2004, 2005-2009, and 2010-2016). These time-stratified models contained an interaction between immigration status and sex.” (methods – lines 251-255)

“The age and sex stratified models revealed utilization of less medical imaging was similar for males and females across most age groupings and imaging modalities (S4 Table). Only a few percentage points differed between the relative rates of males vs females. The largest sex differences were observed in older aged immigrants for CT imaging with males using less than females. Aside from both males and females having higher use of ultrasonography over secular time, no temporal effect was observed when considering the relative rates by immigration period (S5 Table).” (results – lines 330-335)

Regarding the interpretation, these analyses indicate, aside from the very few age-sex/modality scenarios, overall lower use of medical imaging is similar between men and women immigrants when compared with non-immigrants. Furthermore, immigrants have consistently over time underused medical imaging compared to non-immigrants except for ultrasonography which has shown a slight upward trend in the use among immigrants vs. non-immigrants.

“Our sex and immigration period analyses further illustrate no major sex differences in use, and that the disparities seen in utilization of medical imaging have not improved over time. Insights provided by this study support the importance of addressing disparities in healthcare utilization of immigrants, which may be instructive for policy-makers and practitioners.” (discussion -- lines 351-355)

In the Introduction, please add information on how the healthcare system works in Ontario, and the nirn al routed that people take to request/ be referred to receiving medical imaging.

**Response:** We have added how the healthcare system works in Ontario within the community setting. The following sentence was added:

“In the community setting patients must first consult with a general practitioner to obtain a referral for medical imaging.”(lines 128-129)

Please include your findings in a broader perspective (beyond the Ontarian/ Canadian Healthcare system) in your Discussion section.

**Response:** Throughout the discussion section, we have now cited more literature from other jurisdictions and have put our findings into a more international perspective (see added text in the tracked change version of the manuscript).

Some examples include, but are not limited to:

“Evaluating the utilization of radiologic services of immigrants is well-suited to be conducted in Canada. Access to necessary medical care is readily available to all citizens and residents independent of insurance coverage or financial payments for service. Conducting such evaluations in Canada circumvents the financial burdens and systemic challenges often encountered in other countries, providing a more efficient and equitable assessment environment.” (introduction – lines 126-128)

“Evidence from this study provides valuable guidance to healthcare policymakers in regions where immigration plays a significant role in shaping the composition of both current and future populations.” (conclusion – lines 440-442)

**COMMENTS FROM THE REVIEWERS:**

**Reviewer #1:** Thanks for the opportunity to read your manuscript. My role is statistical reviewer, so I have focused on the design, data, and analysis that are presented. I have put general comments first, followed by questions relevant to a specific section of the manuscript (with a page/line reference).

This manuscript examines differences between immigrants and non-immigrants in utilisation of medical imaging. The study uses routinely collected health data from Ontario, Canada and includes all residents registered for health services in this province between 1995 and 2016. This data was linked to a national immigration database. The main analyses used a matched sample of immigrants to non-immigrants, using a pool of potential matches created per year. Matching was based on age at time, sex, and area. Main models comparing utilisation between immigrants and non-immigrants included a measure of comorbidity burden, age, sex, and use of primary care.  The main analysis used recurrent event survival analysis - this is appropriate I agree with the authors that this has advantages over using a generalised linear model with a count distribution. Participants with missing covariate information were retained in the analyses using the 'missing as indicator' approach which assumes that this data is missing-completely-at-random. Several sensitivity analyses are considered, including starting follow-up 2 years later so the same approach to measuring comorbidity is used in both immigrants and non-immigrants, and the use of head CT in participants experiencing a TBI, which is not expected to show a difference between immigrants and non-immigrants.

Using a different temporal window to allow for a lookback period to determine comorbidities in immigrants does not make an appreciable difference to the main results. This is an interesting study - results are well presented, and I think the analysis is good (a few queries below).

P8, L159. So for immigrants, comorbidity status is concurrent with imaging utilisation, while for non-immigrants this precedes measurement of utilisation. Given that healthcare utilisation is driven by need, does this inappropriately adjust for current health status in immigrants and drive estimated differences between immigrants and non-immigrants?

**Response:** This would only affect the first two years of results when the comorbidity score was backfilled for the immigrant group. Indeed, medical imaging is driven by healthcare need which is why we chose to control for comorbidity score given the well-established literature on the healthy immigrant effect. However, comorbidity status was measured to precede the measurement of utilization for both the immigrants and non-immigrants for >2 years of observation. This was why we conducted a sensitivity analysis where we shifted the observation start date to 2 years in the future so that both immigrants and non-immigrants had a calculable comorbidity score using 2-years of data. We do not think that this would lead to differential bias between immigrants and non-immigrants by controlling for this confounding factor. We believe that we may be underestimating the true effect as the results from the multivariable regression table (S6 Table) illustrate an even lower relative rate of imaging for immigrants using non-backfilled data.

P8, L163. What information did you use to estimate that comorbidity status would not change within the first 2 years of arrival?

**Response:** We have used previous literature (Fraccaro *et al.*) to assume that the comorbidity score would not drastically change within the first 2 years of follow up. The supporting reference is a study performed in the United Kingdom over 10-years and ~290,000 individuals which saw only 16% of individuals experiencing a change in comorbidity index. We have added this reference to the sentence (lines 211-214).

The reference used is:

Fraccaro P, Kontopantelis E, Sperrin M, Peek N, Mallen C, Urban P, et al. Predicting mortality from change-over-time in the Charlson Comorbidity Index: A retrospective cohort study in a data-intensive UK health system. Medicine (Baltimore). 2016;95(43):e4973. https://doi.org/10.1097/MD.0000000000004973. PMID: 27787358.

We have also investigated if the assumption was correct in our data. We calculated the average comorbidity score for immigrant and non-immigrant groups within the first 10-years of follow-up (see newly added S1 Fig). The average comorbidity score did not drastically change during the first 2-years of follow-up for the *non-immigrant* group. Thus, we believe that using the prior literature and the trend observed in non-immigrant groups from our data, our assumptions hold, and we can safely use the backfilled comorbidity score. We have added the following text to the methods section to illustrate the satisfaction of the assumption:

“This assumption was confirmed by calculating the average comorbidity score for immigrants and non-immigrants during follow-up which showed no major comorbidity changes in the non-immigrant group within the first 2 years of observed follow-up (S1 Fig).” (lines 211-214)

P6, L117. Were non-immigrants taken out of the pool of possible matches once matched?

**Response:** The reviewer is correct that non-immigrants were removed from the eligible pool of possible matches once matched. We have added “without replacement” to the sentence to provide this clarification (lines 161).

P6, L118. PLOS medicine has an international readership, what size (area and pop) would a typical forward sortation area have?

**Response:** The reviewer makes a valid point. The forward sortation area (FSA) is used by the Canadian postal service for mail delivery and planning. It is approximately proportional to population density and geographic size. Within an urban setting, a single FSA is only a few square kilometres and can have >100,000 individuals. In a rural setting, the opposite is observed. The purpose of matching the FSA at baseline was to provide a geographic measure to adjust for access to care. We have now added the following text to convey that FSA is proportional to population density.

“This geographic measure is used by the federal postal service and is proportional to population density” (lines 164-165)

 P6, L140. Are there any private imaging services in this province that might not be captured by the OHIP?

**Response:** The reviewer raises a valid point. While it is theoretically possible for some imaging to be conducted in private settings not captured by our population-based data sources, we believe such instances are infrequent. We have used the OHIP database, as well as in-patient hospitalization and emergency department records, to capture imaging events. We are confident this approach provides a near-complete picture of medical imaging utilization within the province. To acknowledge this minor omission of privately conducted imaging events we have added the following text to the data sources section of the methods:

“This encompasses all medical imaging performed within the province, except for imaging provided by private practices, which are uncommon.” (lines 190-191)

P8, L177. Does 'accounted' mean that this was achieved as a result of prior matching, or because there was stratification or adjustment when estimating the rate differences?

**Response:** ‘accounted’ refers that this was a result of the prior matching criteria (age, sex, index year, and geographic region). We have now used the word adjusted and have made this explicit that it is due to the matching of baseline matching covariates. The text now reads:

“Rate differences and 95% confidence intervals (95%CI) for each imaging modality outcome were calculated for immigrants relative to non-immigrants and adjusted for baseline age, sex, index year and geographical region due to prior matching.” (lines 230-231)

P8, L186. Were participants censored at death? Was there any way to determine when participants moved from Ontario and no longer at risk?

**Response:** The reviewer is correct that participants were censored at death. Our censoring criteria are defined as “loss of OHIP eligibility (moved out of the province), death, or 31 December 2016 whichever occurred first” in the text. We have now made censoring explicit with sentence now reads:

“Individuals were followed from the index date until they were censored on the loss of OHIP eligibility (moved out of the province), death, or 31 December 2016 whichever occurred first.” (lines 166)

We can determine when participants move out of Ontario and are no longer at risk through information captured by the Registered Persons Database. This provincial database contains the information of all residents in the province who maintain their governmentally provided health insurance plans. Upon moving out of the province, individuals must report this to the provincial government and are thus no longer eligible for the health insurance plan. Thus, we can determine when participants were no longer at risk by determining when they moved out of the province and were no longer eligible for OHIP. We have added the following text to the description of the Registered Persons Database section of the methods:

“Nearly all of Ontario’s 14-million population, including immigrants, are contained in the Registered Persons Database, which is managed by the Ministry of Health and Long-term Care. Upon relocation out of the province, individuals are no longer eligible for health insurance and must notify the Ministry.” (lines 182-183)

P8, L191. How much covariate information was missing from the data? Were those missing covariate data similar to those with complete data? i.e. is it reasonable to assume that this data is missing-completely-at-random?

**Response:** Only two covariates had missing information from the data. In Table 1 we present that approximately 0.2% of socioeconomic status was missing, and <0.01% of immigrant category class was missing. As mentioned in the previous comment, the data for socioeconomic status are obtained from the Registered Persons Database. This dataset is considered a complete capture of resident status in the province given its use for determining eligibility for the health insurance plan. We believe that it is reasonable to conclude that the missing data is similar to complete data given the nature of the underlying data generating process used to populate the Registered Persons Database and the very small % of missing data.

P10, L213. Was this because they left the region?

**Response:** This was because an ADG score could not be calculated. There are likely several reasons why this is the case. The first is that these immigrants did not interact with the healthcare system in a way which would precipitate the calculation of an ADG score throughout the study observation. Additionally, a large number of these immigrants came into the province in the latter period of the study. Approximately 125,000 of these individuals immigrated between 2014-2016 and thus a single ADG score could not be calculated given that our comorbidity score needed 2 years of follow-up for it to be calculated. This has now been made clear in the text:

“A total of 2,165,142 immigrants were identified during the study period; 316,920 were excluded (14.6%) because an ADG score could not be determined due to a lack of interaction with the healthcare system, leaving 1,848,222 immigrants eligible for study inclusion and matched to an equal number of non-immigrants.” (line 273)

P13, L272. I think another word other than 'necessary' could be used here, I am sure many of the imaging services provided other than head CTs were necessary to the participants.

**Response:** We have replaced ‘necessary’ with ‘indicated’ to convey that these head CTs were likely ordered as an indication of the underlying head trauma. The sentence now reads:

“When focusing only on indicated imaging (head CTs for a traumatic head injury), there were no differences in the mean number of CTs between immigrants and non-immigrants (S2 Fig).” (lines 339)

L13, L272. If immigrants presenting to the ED with head trauma are more likely to receive a head CT (26% vs 18%), but the overall number of CTs per trauma is similar between immigrants and non-immigrants, does this mean the distribution of the number of head CTS is different (i.e. non-immigrants more likely to get multiple head CTs)?

**Response:** The reviewer is correct in this statement that there may be differences in the distribution of proportions for imaging exams when analyzed in this manner. However, we note that the population of immigrants and non-immigrants to produce these estimates (S2 Fig) are not the same as the cohort of individuals used in this study. In keeping with the analytical techniques used in our study, this sensitivity analysis was used to illustrate that the average number of imaging for a head CT should not differ for immigrants vs. non-immigrants for this clinical indication.

**Reviewer #2**: Thank you, editor, for sending this paper to read.

This paper raised good research question, well designed methodology and analysis and interpretation. Such findings are expected the people from immigrant's background have to face several countries in the destination countries, and always low service utilization compared to local population.

The utilization of medical imaging is depending on the diseases prevalence. Without knowing patterns of disease, we cannot say that there is disparity of medical imaging services utilization.
As authors mentioned migrants have more healthy migrant effects. Authors should mention in the limitation of the study or provide data for this.

I hope this feedback help to revise this work.

**Response:** To account for the alternate disease states of immigrants and non-immigrants, we have used the Aggregated Diagnosis Groups (ADG) score to calculate an overall measure of comorbidity burden for all individuals in our study which was then controlled for analytically in our multivariable regression models. The calculation of this comorbidity score used diagnostic codes associated with physician billing records, emergency department visits, or hospitalizations within the healthcare system to provide a numerical summary of the overall comorbidity status of individuals. Indeed, the *healthy immigrant* effect has been well documented to illustrate that the health status of immigrants has been shown to differ from those born in the host nation. By allowing for the ADG variable to be a time-varying covariate which was updated annually, we believe we have adequately controlled for the confounding effect of any differences in the overall health between our immigrant and non-immigrant participant groups.

**Reviewer #3:** Reviewer's Report -
Immigrants medical imaging utilization compared to residents in a universal healthcare system Review for PLOS Medicine Manuscript number: PMEDICINE-D-24-00454R1

Summary: This paper presents an empirical investigation as to how the usage of medical imaging healthcare by ‘immigrants’ in the state of Ontario, Canada, compares to residents. The work benefits from a rich administrative data set – with 21 years of data 1995-2016 of complete records for CT, MRI, radiography, and ultrasonography utilisation of patients – with important patient information including information on gender, age, comorbidity which allows for controlling of key explanatory variables. The statistical methodology is appropriate and well-executed – ways of backfilling data etc to deal with data gaps are appropriate, the sensitivity analysis with CT utilisation for traumatic brain injury is very nice. The Tables and charts are well presented. I have some concerns about how some aspects of the paper are described – and have additional suggestions to improve the contextual understanding and presentation of the work for an international readership – outlined in this peer- review.

Overall, I see the merit in the research, and recommend revisions of the content and presentation of the work undertaken which where address can allow for the paper to progress towards publication in *PLOS Medicine*.

***Title/Abstract*** – Throughout this paper (and even the labelling on your charts!) I do not agree with the distinction between the groups as immigrants versus residents of Canada. The immigrant groups do in fact appear to be residents – so surely it is more accurate to describe the groups as native versus non-native residents of Canada – from what I gather they are distinguished by country of birth i.e. nationality.

**Response:** We agree that the immigrant groups are indeed residents as well. We thank the reviewer for suggesting the use of ‘native’; however, that wording in Canada is typically reserved for our Indigenous communities. Thus, throughout the entire text, figures, and tables we have replaced all instances of ‘residents’ with ‘non-immigrant’ to make the distinction that these group of individuals were not migrants. We believe this to be the most accurate way to describe the two groups of individuals in our study.

The reference to matching 1 to 1 on residents based on age, sex, **immigration year (?)**, and geography is confusing – how can you match native residents versus immigrant residents based on immigration year – this does not make sense to me at least on first read? Please clarify if this is a typo or something else?

**Response:** We apologize for any confusion this may have caused. By matching at the time of immigration year, we are controlling for temporal characteristics at the observation start date. This wording was intended to convey that at the time of immigration, an immigrant was matched to a non-immigrant on the specified matching criteria. For example, a female 32-year-old immigrant who migrated in 2000 was matched to a female, 32-year-old non-immigrant in 2000, from the same geographic region. This ensured that the immigrant was not mismatched to a non-immigrant 32-year-old female from 1995.

We have made the following changes in the abstract and main methods text:

“A total of 1,848,222 immigrants were matched 1:1 to non-immigrants in the year of migration on age, sex, and geography.” (abstract)

“In the year of their migration (observation start date), immigrants were randomly, individually matched to non-immigrants of Ontario one-to-one without replacement on age, sex, and geography.” (methods – lines 160-162)

The results accord with expectations apart from the result on the longer the time since immigration the larger the gap – interesting and slightly counterintuitive.

**Response:** We agree that our results showing a divergence in utilization rates as time elapsed are counterintuitive and have not been observed in prior studies. We hope that our manuscript may be used to guide future research aimed at unpacking this unexpected result.

Keywords- I don’t think the use of emigrants under this paper is appropriate – also I do not think this is a traditional cohort study – so this terminology is misleading (?)

**Response:** We have removed “emigrants” from the keywords as suggested. Our design is indeed a cohort study (retrospective) which has used exact matching at the time of immigration to reduce confounding.

Introduction - please provide clarification on your resident and migrant distinction.

**Response:** Now that we have referred to our groups as ‘immigrant’ and ‘non-immigrant’ throughout the entire text/title, the distinction between those who are born in the country and those who immigrating is clear.

Since Canada is a universal healthcare system in which GPs are the main gatekeepers – and GPs can be a key route through which patients access medical imaging, it is important to also cite literature which examines immigrants access to GP services – e.g. Barlow, Mohan, and Nolan (2022) Utilisation of healthcare by immigrant adults relative to the host population: Evidence from Ireland. *Journal of Migration and Health*.

**Response:** We have now outlined the immigrant use of primary care providers. We thank the reviewer for providing this reference and have used it in our citation. We have added the following text to the introduction which outlines the experiences of immigrants utilizing general practitioner services.

“Immigrants must also acquire a general practitioner in their new host nation, and many report difficulties finding one, often resulting in the underutilization of primary care”. (lines 117-118)

Line 85: radiology **use or access**

**Response:** We have clarified that it is radiology “**use**” and have made that distinction within the text at the stated line (line 120).

Line 87: crucial to gain insight into any disparities in immigrant populations for **policy making and healthcare planning**… Please refer to important policy documents from the WHO in this area e.g. World Health Organization, 2019. Promoting the Health of Refugees and Migrants Draft Global Action Plan, 2019–2023. <https://apps.who.int/gb/ebwha/pdf_files/WHA72/A72_25-en.pdf>.

**Response:** We thank the reviewer for providing this reference document and have referenced a [similar WHO published report from 2023](https://www.who.int/publications/i/item/9789240067110). The amended sentence with updated references now reads:

“Thus, it is crucial to gain insight into any disparities in immigrant populations for policy-making and healthcare planning and to ensure equity in the delivery of medical imaging” (lines 122-123)

It would also be good to know if the Canadian government or a Canadian Medical Association – or locally in Ontario had policies published on migrant health – there is no enough discussion of policies and planning in this area – particularly if there is any consideration of this in terms of radiology which is a unique selling point of your paper – you do not sufficiently sell the special case of native v non-natives use of medical imaging as a motivation for your research – the use or non-use of this aspect of healthcare obviously has downstream effects e.g. lack of detection/diagnosis leads to poorer patient outcomes (more advanced cancers etc), preventable mortality etc . – provide references to strengthen your arguments. Greater sell the novelty and wider importance of this work.

**Response:** We have added the following paragraph to the introduction which outlines the importance of studying medical imaging in immigrant groups.

“Frameworks for healthcare use and health-seeking behaviours in immigrants have been well-described [9,10]. Health systems and governmental policies provide macro-structural factors for immigrants, while the delivery style of care in the host jurisdiction, such as volume and costs, contribute to utilization [9]. Immigrant-specific factors, such as the degree of assimilation or cultural beliefs about health, influence an immigrant's propensity to seek care and their perceptions of the need for services [10]. Furthermore, the length of time an immigrant has resided in the host country can positively influence their health-seeking behaviours, as longer duration is often associated with increased familiarity with the healthcare system and greater social integration [9]. Understanding the use of medical imaging among migrant groups is crucial for preventing downstream consequences such as late disease detection or poor health outcomes [8,11,12].” (lines 105-114)

The work also does not provide much consideration of the world beyond the Canadian context – how generalisable are the findings? What lessons can be gleemed for other countries/regions and jurisdictions?

**Response:** Like feedback received from the editorial team and the below comment, we have now placed the results of the work into a more international perspective. See changes in the tracked version of the manuscript throughout the introduction and discussion sections.

You do not provide a clear research question – no outline a clear aim of the research. The hypothesis you provide is not supported by any other empirical references or a previous theoretical underpinning – this is needed to strengthen your argument. – Is it an assimilation or integration argument that you are making – the longer a person is resident in a country they adopt the norms of the local population, including healthcare utilisation norms?

**Response:** We have now made this clear by explicitly stating the research aim, which was to evaluate the medical imaging utilization of immigrants using different imaging modalities.

“This study aimed to evaluate the health services utilization of medical imaging in immigrants and examined the use of computerized tomography (CT), magnetic resonance imaging (MRI), radiography, and ultrasonography spanning two decades for immigrants within a universal healthcare system. Our objective was to determine the association between immigration status and medical imaging utilization, and the extent to which this association differs by the age at which individuals immigrate and the length of time since immigration.” (lines 137-138)

Our hypothesis on a longer residency and similar usage in healthcare is now supported by providing literature on the assimilation of immigrants in the introduction. This was also brought up by Reviewer #4 and addressed below.

And then in testing this hypothesis I see later you have categories of >10 years in Canada, > 20 years etc – is the hypothesis based on any particular cut off period (e.g. over 10/20 years) or just a gradient over time should be seen?

**Response:** These cutoffs are not based on any empirical evidence but rather a gradient over time was expected.

***Methods***

You have really comprehensive coverage on migrants. I am uncomfortable with the use of the term ‘landed’ throughout – maybe arrival/taking up residency is a better way of phrasing this?

**Response:** The use of the term ‘landed’ aligns with the official nomenclature used by Statistics Canada for referring to the official date an immigrant has arrived within the country (<https://www23.statcan.gc.ca/imdb/p3Var.pl?Function=DEC&Id=103339>). We believe that we should remain consistent with the nomenclature used.

It is not clear whether or not you have the exact country or region of origin of migrants in your dataset – if not, please clarify (maybe I have missed this) – the literature has shown that there can be significantly heterogenous findings on healthcare utilisation by different migrant groups – and you are treating them all as a homogenous group. For example, Mohan (2021) The influence of caregiver's migration status on child's use of healthcare services: evidence from Ireland, *Sociology of Health and Illness,* finds different patterns in use of healthcare services by children of different migrant groups e.g. those from less advanced non-English speaking non-European countries availed less of GP services less than native population – and other types of migrant groups. This paper may also be relevant for commentary on your 0-19 year old group – since children (of migrant background) rely on their parents/caregivers to help them access (and are involved in the decision making process around) healthcare services – and the parents are likely to be of a migrant background themselves.

**Response:** From the Immigration, Refugees and Citizenship Canada database we would have country of origin captured and this is stated in the methods. However, members of the research team met and concluded that further stratifying and incorporating the region of origin for the migrants would add additional complexities to an already densely packed manuscript. We do believe that there is heterogeneity in the healthcare utilization between immigrants of varying ethnic origins which warrants further investigation. This was highlighted in our discussion section as a limiting factor of the current body of work.

“Lastly, we did not consider race or ethnic origin, and thus could not determine if these factors influenced utilization rates. Previous research from the U.S. has illustrated that diagnostic imaging utilization differs by patient race and ethnicity” (lines 433-435)

You may need to better describe in the Introduction the typical pathways for a patient to access medical imaging services. For example, describing the GP as gatekeeper role – which I assume is free at the point of use (or is there any co-payments) and then if there are co-payments in services – the reason I query this here is you say that a source of your medical imaging data is from fee-for-service physician billing records (OHIP Schedule of Benefits and Fees) – and an international readership will not understand what this is under a universal healthcare system. In the UK’s NHS which is a universal healthcare system there are no fees for services – e.g. copayments – so outlining the institutional setting is important.

**Response:** Like the comment provided by the editorial team, we have added details on how a patient may access medical imaging within the community to the introduction by first consulting with a general practitioner.

We state in our introduction that “Access to necessary medical care is readily available to all citizens and residents independent of insurance coverage or financial payments for service” (lines 125-126) to convey that there are no copayments or other fees associated with medical care. When a physician performs any medical procedure or consultation, the provincial government is billed for the service provided; hence, the OHIP database collates these records. We have now made this clear within the text:

“Medical imaging data were captured through linkages to fee-for-service physician billing records to the Ministry of Health from the OHIP database, inpatient hospitalization imaging from the Canadian Institute for Health Information’s Discharge Abstract Database, and emergency department imaging from the National Ambulatory Care Reporting System.” (lines 187-188)

Please define meaning of ICES.

**Response:** ICES is the formal name of the organization where the analyses were conducted. In 2018, the institute formerly known as the Institute for Clinical Evaluative Sciences officially adopted the initialism ICES as its official name. This change acknowledges the growth and evolution of the organization’s research since its inception in 1992, while retaining the familiarity of the former acronym within the scientific community and beyond.

Is your control variable of a visit to a primary care provider a binary variable – and is that in the prior 12-month period of what is the exact definition?

**Response:** Rostering to a primary care provider was recorded as a binary variable. We have now made this explicit within the text. This was measured using a look-back window in the previous 2 years to record whether that individual was rostered to a primary care provider or not in the past. Since this variable was updated annually, it provided a rolling window which captured patterns of being rostered to a doctor over time which would influence prescribing patterns for medical imaging depending on patient needs. The sentence now reads:

“Access and rostering to a primary care provider were collected using a 2-year look back window to determine the presence or absence of being rostered to a general practitioner. This served as a proxy to account for access to healthcare, as this may confound imaging rates given primary care physicians act as gatekeepers to the healthcare system.” (lines 215-216)

***Discussion***

Your surprising result which is nice that you can test in the data that you have (many studies do not have duration of residence in the country) – was that the longer a person is resident in Canda there medical imaging use remained lower than that of their Canadian counterparts – more could be commented on this again bringing in theory or supporting references on lack of assimilation/integration. The point you make that ‘passively waiting for access for new immigrants to improve over time does not occur’ is a key indication from this paper – to which more could be made of this in terms of evidence gathering to inform national government policies and the like of supranational organisations such as the WHO, UN, EU etc.

**Response:** We have added the implications of this indication by amending the text to read:

“This suggests that passively waiting for access for new immigrants to improve over time does not occur and highlights an opportunity for interventions targeted at bolstering health literacy among migrants” (lines 378-379)

Additional changes can be viewed throughout the discussion (see tracked changes).

The point on older migrant groups accessing services less is also insightful – you do not have data on whether they have children or not – but again the family circumstances of the household are likely to be key here (as in the case of children previously mentioned) – so while you can’t answer this question it may be that older immigrant people with children may be better able to access services if there is a language barrier etc. – as the children can speak on their behalf – i.e. there may even be heterogenous access within this group depending on family circumstances.

**Response:** We have now added that family dynamics, particularly within the older-aged immigrants, may influence the utilization of healthcare by facilitating any language barriers. We have added the following sentence to the limitations

“Family composition was not captured, and it may be possible that children of older immigrants or other familial members can serve as aids in any language barriers experienced when navigating the healthcare system and influence imaging use” (lines 427-428)

A reason for lower utilisation of services by immigrant groups which has not been mentioned is that sometime immigrant groups go back to their home country of origin of care e.g. Wallace et al (2009) showed that Mexican migrants in the US went home to Mexico to receive care – this may not be to the same extent in the Canadian setting.

**Response:** In response to the reviewer's comment, we have added a sentence to the discussion acknowledging the potential for return migration for healthcare and thank them for the reference. While this may be less common in Canada, its inclusion strengthens the international context of our work, as suggested in a later comment.

“Immigrants may also return to their country of origin to seek medical care [51], although this is unlikely within our universal healthcare system and more prominent in other jurisdictions with alternate healthcare systems.” (lines 366-369)

Again, the policy and practice implications and the relevance for an international audience needed greater consideration.

**Response:** We have now addressed the policy and practice implications of the work throughout the introduction and discussion sections (see manuscript with text added tracked version). We have also expanded our references to cite literature from other jurisdictions around the world.

Overall, great data and well executed analysis which could be valuable where the paper is strengthened.

**Response:** We thank the reviewer for their insightful feedback and believe incorporating the suggested changes has significantly strengthened the manuscript.

**Reviewer #4:** I wish to congratulate the authors in undertaking this ambitious study that illustrates diverging patterns of imaging completion among immigrants compared to non-immigrant groups. The data and modeling are well done, but its ability to be interpreted depends on how it's contextualized. As such I feel that the paper could benefit from better framing and slightly more analysis.

First, it's a bit unclear why, given the author's well-placed literature about disparities in radiography and its multifaceted drivers why the authors' hypothesis is that in Canada disparities would decrease with time in Canada. Do they presume that in Canada the limiting factor is unmeasured system navigational skills?

**Response:** As per the comment below by the reviewer to incorporate a conceptual model of immigrant healthcare utilization to support the introduction/discussion, we have now explicitly stated how a longer residency in a host nation is predictive of increased healthcare usage. This has now further supported the reasoning for our original hypotheses that assimilation and the adoption of healthcare usage would trend towards the behaviours of individuals born in the host nation. We have added the following to the introduction paragraph where we discuss the current framework on length of residency:

“Furthermore, the length of time an immigrant has resided in the host country can positively influence their health-seeking behaviours, as longer duration is often associated with increased familiarity with the healthcare system and greater social integration” (lines 109-112)

Related to the above point, it is important to emphasize what the authors have identified is imaging *completion*, which as they hint in the discussion, is essentially a function of differences in health care access, clinic/inpatient utilization, provider driven ordering, patient acceptance, and patient follow-through. The universal health system in Ontario provides good access to care and while there are very wide post-match disparities in clinic visits as seen in Table 1, ideally that is well controlled for in models. I assume the authors don't know about uncompleted orders, although I interpret the fact that overall imaging rates only diverge after more time in Canada as arguing against systematic cultural resistance to imaging.

**Response:** The reviewer is correct that through our study design and analytic approach, we have sufficiently controlled for access to care (through geographic matching and time-varying socioeconomic positioning) and access to a physician (through being rostered to a doctor) which were time-varying covariates. The reviewer is correct that we are observing medical imaging **completions**, and that prescribed but *uncompleted* imaging orders would go undetected. Unfortunately, we do not know the number of imaging orders prescribed and then filled. This would have provided much richer contextual evidence to both prescribing patterns within the healthcare system, as well as the health-seeking behaviours of our immigrant groups. We have added this as a limitation.

“Additionally, we were unable to account for imaging prescribed in outpatient settings but not ultimately performed.” (lines 423-424)

However, there is little interrogation about whether providers are choosing to offer radiological tests to older immigrants in lower amounts in outpatient settings (presuming that outpatient ordering accounts for a substantial amount of imaging in this sample), particularly as there is substantial literature on provider-driven discrimination in ordering.

**Response:** Similar to the point above, the reviewer raises a valid point that this may be a contributing factor driving the disparities observed across the age groups of immigrants. However, given the population-based nature of the data, we do not have the level of granular data in this study to ascertain general practitioner prescribing preferences for radiological tests in the outpatient setting and if these orders were filled or not. As previously mentioned, we have stated this as a limitation of the work.

I think that this paper would be strengthened by 1) a conceptual model (which I strongly recommend) and 2) a sensitivity analysis to investigate discrimination in provider ordering. As for models, there are several out there, but any model should at the very least undergird the importance of the question and support your hypothesis that there is convergence in imaging rates with time. Having a model (or at least explicitly delineating challenges along the patient need to imaging completion pipeline) might also help frame paragraph 2 and 3 of discussion explaining this gap.

**Response:** We thank the reviewer for this suggestion and have incorporated the following conceptual frameworks and have referenced them in the introduction:

- Peng B, Ling L. Health service behaviors of migrants: A conceptual framework. Front Public Health. 2023;11:1043135. https://doi.org/10.3389/fpubh.2023.1043135. PMID: 37124818.
- Yang PQ, Hwang SH. Explaining Immigrant Health Service Utilization: A Theoretical Framework. SAGE Open. 2016;6(2):215824401664813. https://doi.org/10.1177/2158244016648137.

This has now highlighted the research importance and the underpinnings of immigrant health services utilization. This has strengthened the presentation of the current state of the literature and bolsters the objectives of the work. (see newly added introduction, paragraph 2).

As for the second point, it would be difficult to perform a sensitivity analysis to investigate discrimination in provider ordering given the current limitations of the population-based administrative data. This work may be well suited to be performed within the hospital and/or clinic setting where data may be more granular rather than at the administrative billing level.

As for the second item, I understand that the team does not have detailed indication data, but it seems they do have ICD or similar diagnoses. It would be useful to complement the Emergency Room CT Scan analysis with a sensitivity analysis that identifies imaging rates for a diagnosis attached to a common incident complaint (e.g., CT/MRI head for incident headache over 65, a "red flag"). This example isn't prescriptive, of course, but simply an example of what the authors may attempt.

**Response:** The reviewer is correct that we do not have detailed indication data for the individuals within our cohort. We have recognized this as a limitation within the appropriate Discussion section. While we appreciate the suggestion, incorporating additional analyses on common complaints is beyond the scope of this current work, as we have already conducted a sensitivity analysis demonstrating no difference in the average number of CT scans for an indication where we did not expect to see differences. However, our research team recognizes the value of future research incorporating detailed clinical indication data to further unpack the results observed in this study.

To encourage this, we have added the following to our limitations section:

“The indication for imaging was not captured, thus it was not possible to determine if the imaging was necessary. Future research on imaging utilization in immigrant groups should consider detailed clinical indications for imaging to elucidate the specific reasons driving the observed utilization rates.” (lines 425-427)

Finally, it would be useful, even as a supplementary table, to include an interaction with sex to your Table 2 Model, to identify intersectional gender-immigration disparities, which are probably quite important.

**Response:** We thank the reviewer for this insightful suggestion and agree sex considerations should be considered to aid in identifying the intersectionality of gender-immigrant disparities in medical imaging utilization. We have performed an *ad hoc* analysis where we have stratified our multivariable regression models by both age at immigration and sex to explore this association. This has now been added as S4 Table in the supplement.

We show that there are some instances where the utilization of medical imaging does differ by sex, but this was only observed in the older age groups for CT imaging. However, these associations are not consistent across all ages and sexes. Additionally, as per the comments from the editorial team above, we have also performed analyses where we have evaluated imaging utilization of our cohort by both sex **and** immigration era to evaluate if disparities have changed over time. The added text can be seen in the response to the editors above.

***Several minor issues:***

Page 7, line 146: The sentence "Utilization for CT, MRI, radiography, and ultrasonography modalities were evaluated daily for all examination types". Some clarification is needed by "evaluated daily". If you're simply prefacing the deduplication efforts, you might consider removing the word "daily" above to avoid confusion.

**Response:** The reviewer is correct that we were prefacing the deduplication efforts used. As suggested, we have removed the word “daily” (line 194).

Page 13, line 267: "Older adult immigrants had slightly higher RR of ultrasonography" (RR, 0.98; 95%CI: 0.98, 0.98). The RR is less than 1—it should be "lower".

**Response:** We thank the reviewer for noticing this. We have corrected the text to “lower” (line 328).

Page 15, line 312-314: I presume "increased" should be "increase". Please also refer to Table 2 again here. Also, this hypothesis should be easily proven by disaggregating by sex, if not ultrasound type?

**Response:** The reviewer is correct that this should be increase and not increased. We have also split the sentence in question into two to make comprehension easier. It now reads:

“Detailed data on indication were not available, however, we observed that the largest increase in ultrasound use occurred in young adults of reproductive age. Similarly, the highest utilization rates were within the earliest time-period from the arrival date.” (lines 389-390)
