# Supplementary material for: Medical imaging utilization in migrants compared with nonmigrants in a universal healthcare system: A population-based matched cohort study
Source: PLoS Med. 2024 Oct 22;21(10):e1004474. doi: 10.1371/journal.pmed.1004474 (PMC11495850; doi:10.1371/journal.pmed.1004474)
Supplement: Attachment — Submitted filename: Response_to_reviewers R3.docx [file pmed.1004474.s012.docx]

**Comments from the Academic Editor and editorial Team**

Thank you very much for your revised manuscript. We all agree that the manuscript is much strengthened.

1. The academic editor and the editorial team are interested in seeing interactions across having PCP or not and income quartile. We don't think simple adjustment in a model is sufficient to address how these complex variables interplay with access to healthcare.

**Response:** As immigration status was the primary exposure, we aimed to estimate the overall association between immigration status and the event rate. We thus decided *a priori* to adjust for PCP and income quintile since we believe they are possible confounders.

Further examining interactions between PCP and income quintile will not meaningfully change the overall estimate of the main exposure; moreover, for this work, we did not aim to examine how the association between immigration status and the event varied by PCP, or varied by income quintile. This is an interesting question but was not part of our original aim. We have amended the following in the discussion to highlight this as a need for future work.

We have highlighted this within our discussion: “To account for these barriers to access in our analysis, we adjusted for socioeconomic status and visits to a primary care provider, the gatekeepers to the medical system, yet differences persist. Migrants continued to have lower rates of imaging after considering these proxies for barriers to access. This suggests a need for future research to explore other mechanisms which may aid in explaining the lowered utilization of medical imaging or if these factors are effect modifiers in the utilization.”

2. The academic editor also commented that they would still be interesting for you to frame your results with something like the Levesque framework for access to healthcare.

**Response:** We have now structures and reframed our discussion incorporating the Levesque framework for access to healthcare. Changes can be found throughout the discussion in the tracked changes version of the manuscript. For example, newly included text includes:

“An unexpected finding was that the length of time since migration resulted in a larger difference in imaging rates between migrants and non-migrants, rather than a dissipation of differences over time. Our sex and migration period stratified analyses further illustrate no major sex differences in use, and that the disparities seen in utilization of medical imaging have not improved over time. Insights provided by this study support the importance of addressing disparities in healthcare utilization of migrants, which may be instructive for policy-makers and practitioners [23,39,40]. Thus, it is essential to place our findings within Levesque et al. multidimensional framework of healthcare access which encompasses approachability, acceptability, availability/accommodation, affordability, and appropriateness [41]. Each of these dimensions plays a critical role in shaping how populations interact with the healthcare system.”

“Landed migrants must navigate a new healthcare system and common themes of language barriers, lack of information, and cultural differences may act to impede interactions with the healthcare system and lead to lower healthcare services utilization [15]. Migrants also report a barrier to healthcare access given the need to adjust to a new healthcare system [50,51]. These barriers may hinder migrants’ perceptions of healthcare acceptability given cultural/social factors or the approachability of the new host nation’s healthcare system in their ability to seek care.”

“To account for the barriers to access in our analysis, we adjusted for socioeconomic status and visits to a primary care provider, the gatekeepers to the medical system, yet differences persist. Migrants continued to exhibit lower imaging rates after considering these proxies as barriers to access. This suggests a need for future research to explore other mechanisms that may aid in explaining the lowered utilization of medical imaging or if these factors are effect modifiers in the utilization. Within Canada’s universal healthcare system, migrants are not required to have insurance coverage or pay for medical imaging. However, indirect costs such as travelling to healthcare centres may contribute to the affordability of migrants seeking medical imaging.”

“Migrants have a lower utilization of CT, MRI, and radiographic medical imaging. A longer length of time since migration resulted in a larger negative difference in utilization rates. This observation highlights an important discrepancy in imaging utilization in a universal health system where disparities in healthcare are unexpected. Addressing disparities in migrant medical imaging use requires a multidimensional lens incorporating both healthcare system factors and patient-level perspectives regarding access and beliefs so that strategies can ensure equitable access to healthcare for migrant populations. Evidence from this study provides valuable guidance to healthcare policymakers in regions where migration plays a significant role in shaping the composition of both current and future populations. Future research should explore the reasons for this disparity and whether they impact patient health outcomes, particularly within a universal healthcare system.”

3. We also like to suggest you use the word “migrant” rather than “immigrant”.

**Response:** We now use “migrant” instead of immigrant throughout the text and associated tables/figures when referring to the two exposure groups of individuals in our study.

4. Data Availability Statement: please revise your statement, as authors cannot be the contact person. I also like to encourage you to make the code openly available, you may wish to deposit this in Github or another repository. If code cannot be shared due to legal or ethical reasons then authors should state this in the Data Availability Statement and give details of how to request access to the code (please note that once again, authors can not be the contact person). You can find more information here: <https://journals.plos.org/plosmedicine/s/materials-software-and-code-sharing>

**Response:** The provided contact is not a person but rather a generic email address used by the Data & Analytic Services (DAS) department of ICES. We have amended the Data Availability Statement to make it clear that the email originally provided is not a person. We have also noted that code cannot be shared publicly and that those who meet certain criteria may be granted access to the code by contacting the provided email address. The section now reads:

“The dataset from this study is held securely in coded form at ICES. While legal data sharing agreements between ICES and data providers (e.g., healthcare organizations and government) prohibit ICES from making the dataset publicly available, access may be granted to those who meet pre-specified criteria for confidential access by contacting Data & Analytic Services, available at www.ices.on.ca/DAS (email: das@ices.on.ca). The full dataset creation plan and underlying analytic code used to generate results cannot be made publicly available due to privacy and legal concerns. Those who meet pre-specified criteria may be granted access to the code by contacting ICES Data & Analytic Services, understanding that the computer programs may rely upon coding templates or macros that are unique to ICES and are therefore either inaccessible or may require modification.”

5. Figures: I noticed you use red and green in your figures. Please consider avoiding the use of red and green in order to make your figure more accessible to those with colour blindness.

**Response:** A colourblind-friendly colour palette is now used in all figures.

6. PLOS Style: Discussion layout. Please present and organize the Discussion as follows: a short, clear summary of the article's findings; what the study adds to existing research and where and why the results may differ from previous research; strengths and limitations of the study; implications and next steps for research, clinical practice, and/or public policy; one-paragraph conclusion. Please remove all other sub headers.

**Response:** Sub-headers have been removed from the Discussion.

7. Supplementary materials. This is a kind reminder that supplementary materials are not checked and will be posted as supplied by the authors. Therefore, please double check. Please cite your Supporting Information as outlined here: <https://journals.plos.org/plosmedicine/s/supporting-information> - Please note you may use almost any description as the item name of your supporting information as long as it contains an "S" and number. For example, “S1 Appendix” and “S2 Appendix,” “S1 Table” and “S2 Table. Please ensure each supplementary material has a call out (link) from your main manuscript.

**Response:** Supplemental materials have been checked and all now abide by the provided guidelines as outlined by the provided weblink.

**Comments from the reviewers**: 

Reviewer #1: Thanks for the revised manuscript and responses to my original review. The updated manuscript resolves my original queries, this is an interesting study that I enjoyed reviewing. 
I agree with the authors that excluding missing data when the amount is ~0.2% is reasonable for socioeconomics and immigrant category.

**Response:** We thank the reviewer for their constructive and helpful comments which have strengthened the manuscript.

Reviewer #3: The authors have adequately addressed the comments of my previous review - based on my own assessment of the paper, I recommend the revised version of the manuscript for publication 

**Response:** We thank the reviewer for their constructive and helpful comments which have strengthened the manuscript.

Reviewer #4: I appreciate the authors taking the time to revise their report according to recommendations. A few further items to consider:

1) Page 9, line 157: I would not consider matching to be random process--the probability is being chosen is uniform only given observed characteristics, not unobserved ones. I would remove the word "randomly'.

**Response:** We have removed the word “randomly” from the sentence, as suggested.

2) The Anderson-Gill model is a type of survival analysis, and estimates should be reported as hazard ratios. (<https://academic.oup.com/ije/article/44/1/324/654595>).

**Response:** We thank the reviewer for providing this excellent reference. However, it is very important to note that even in the simple setting of a single time-to-event framework examined with a Cox model, the hazard function is itself a rate function, meaning the hazard ratio is indeed a relative rate (reference: Sutradhar R, Austin PC. Relative rates not relative risks: addressing a widespread misinterpretation of hazard ratios. Ann Epidemiol. 2018 Jan;28(1):54-57. doi: [10.1016/j.annepidem.2017.10.014](https://doi.org/10.1016/j.annepidem.2017.10.014)).

Similarly, under a recurrent event framework examined with an Andersen-Gill model, the regression is modeling the event rate (left hand side of the regression equation), and provides an estimate of the relative rate. This terminology is mathematically accurate (please see Table 1 of the statistical paper published in Statistics in Medicine by senior author Richard Cook: Zhong Y and Cook RJ (2021), Semiparametric recurrent event vs time-to-first-event analyses in randomized trials: Estimands and model misspecification, Statistics in Medicine, 40 (16): 3823-3842. doi: [10.1002/sim.9002](https://doi.org/10.1002/sim.9002))

3) Lines 366-369: Rather than simply say that it is unlikely that immigrants are getting imaging outside of Canada, I would consider offering up the fact that you are matching on 2-year primary care utilization; if they are seeing the doctor in similar amounts in country they should be getting imaging in country in similar amounts as well, theoretically. (Getting additional healthcare/imaging while traveling/living abroad--as many elderly immigrants with regularized immigration status do--is still possible, but less likely).

**Response:** We thank the reviewer for this feedback and agree that through the act of controlling for primary care access, imaging should theoretically be in similar amounts. We have now made it clear within the text which reads:

“Migrants may also return to their country of origin to seek medical care [51]. However, this is unlikely within our study since we controlled for primary care access and may be more prominent in other jurisdictions with alternate healthcare systems.” (lines 378-79)
